# Revisiting differential expression analysis: An updated six-dimensional comparative study

**DOI:** 10.1371/journal.pone.0344709

**Published:** 2026-08-26

**Authors:** Jianxiong Wu, Shaoke Lu, Hui Yao, Zhaoyuan Fang

**Affiliations:** 1 Department of Colorectal Surgery and Oncology of the Second Affiliated Hospital, Centre of Biomedical Systems and Informatics of Zhejiang University-University of Edinburgh Institute (ZJU-UoE Institute), Zhejiang University School of Medicine, Zhejiang University, Hangzhou, Zhejiang, China; 2 Edinburgh Medical School, College of Medicine and Veterinary Medicine, The University of Edinburgh, Edinburgh, United Kingdom; Karmanos Cancer Institute, Wayne State University School of Medicine, UNITED STATES OF AMERICA

## Abstract

Differential expression (DE) analysis is probably the most prevalent task for transcriptomic studies. However, recent technological advances have seen a revival of methodological interest in DE algorithms. In this study, we performed a comprehensive updated comparative study of 12 representative DE methods using 80 simulated and real datasets. We assessed the adaptability of these methods across varying sample sizes and diverse data scenarios. This evaluation compiled a six-dimensional overview of key properties: detection accuracy, sensitivity at a low false discovery rate, false positives, stability, robustness to outliers, and robustness under noisy conditions. Strikingly, no single methods outperformed others across all evaluation criteria and sample sizes, emphasizing data-specific and scenario-specific method choice. At the widely adopted small-sample size of *n* = 3, ABSSeq generally outperformed other methods. As sample size increased to *n* = 5, the sensitivity of DESeq2 and two edgeR v4 algorithms (QLF slightly better than LRT) also raise up under a stringent false-positive control. DESeq had even fewer false positives than DESeq2, at the price of reduced sensitivity. In terms of robustness, Wilcoxon and ROTS are robust to noises for small sample sizes. Moreover, Wilcoxon is also robust to outliers, together with several other methods (ABSSeq, voom, and T.test). NBPSeq and most methods had a good stability even at small sample sizes, except three methods (ROTS, DSS, and T.test). For larger sample sizes (*n* > 30), all methods performed much better. Finally, we provided a “BaGua (eight trigrams)” map summarizing the multi-dimensional performances of methods, as well as a tree diagram guiding practical method selection. Together, this study outlines a systematic and updated benchmarking framework for DE analysis, emphasizing a balance between accuracy and consistency.

## Introduction

RNA sequencing (RNA-seq) is a gold standard for high-throughput quantification of genome-wide expression. The generated transcriptomic datasets contain rich information for subsequent biomedical discoveries. To narrow down to a relatively small set of testable genes, it is inevitable to employ differential expression (DE) analysis [1], either standalone or in combination with dimension reduction, clustering, and functional analysis (such as pathway enrichment and network inference). Moreover, recent transcriptomic technologies such as single-cell RNA-seq (scRNA-seq) [2], spatial transcriptomics [3], and third-generation long-read RNA-seq [4], further reinforcing the central role of DE analysis with respect to prominent noises, sparsity, and limited samples [5–8].

Although this field has seen the development of numerous methods [9–12], many biological researchers are often satisfied with a single run of a single method, relying on the so-called popularity of methods rather than scenario suitability, as well as assuming that DESeq2 or edgeR would perform well on any data generated under any conditions. Clearly, an updated and comprehensive benchmarking of representative DE methods, highlighting their respective strength and weakness, summarizing their suitable use scenarios, remains highly warranted.

To accomplish such a task, we first briefly overview the representative methods in DE analysis. Broadly speaking, these methods are either parametric or non-parametric [1]. The parametric methods mostly rely on the negative binomial (NB) distribution, such as DESeq2 [13], DESeq [14], edgeR v4 [15–17], NBPSeq [18], ABSSeq [19], and DSS [20], diverging mainly in the dispersion estimation strategies, which critically influence their performance. Fewer parametric methods, such as limma-voom [21] and ROTS [22], rely on the assumption of near-normally distributed data. On the other hand, non-parametric methods, such as Wilcoxon [23] and NOISeq [24], do not rely on the distribution assumptions, with less restricted application to diverse data.

Previous evaluation work already reveal several useful points. Williams et al. [25] demonstrated that among the three main steps in RNA-seq analysis—alignment, quantification, and statistical testing—statistical analysis has the greatest impact on performance. In addition, Robles et al. [26] and Ching et al. [27] suggested that, under a fixed budget, increasing the number of biological replicates is more beneficial than increasing sequencing depth. Furthermore, Assefa et al. [28] found that many DE methods perform poorly when analyzing low-abundance lncRNAs and mRNAs. Recently, Li et al. [29] discovered that DESeq2 and edgeR often fail to properly control the FDR in population-level RNA-seq studies.

Building upon these works, we aim to provide a solid and conclusive benchmarking for those most representative methods, including more recent methods not previously evaluated. We proposed a more systematic evaluation framework of six metric dimensions, including predictive performance, sensitivity at low false positive rates, control on false discoveries, two types of robustness, and stability. We specifically emphasize reproducibility (robustness and stability), as an important quality that an excellent, generalizable method should demonstrate. Furthermore, we extended the evaluation over a large number of real and simulated datasets. Finally, we constructed a standardized multidimensional performance “BaGua (eight trigrams)” map and practical method-selection guidelines to provide researchers with suitable DE methods for different sample sizes, perturbation characteristics, and analytical objectives.

## Materials and methods

### Overview of differential expression methods

In this study, we systematically evaluated 12 differential expression analysis methods. All calculations were performed in R 4.3.3, and the parameters of each method were set to their default values (see the supplementary materials for specific versions and parameter settings). The normalization choice was also kept as the default for each method, without separately evaluating its effect [28,30,31].

DESeq2 employs a NB generalized linear model (GLM). It estimates dispersion for each gene using maximum likelihood and then fits a smooth curve to model the relationship between mean expression and dispersion. Empirical Bayes shrinkage then pulls gene-wise dispersion estimates toward the fitted trend to reduce noise. The shrinkage level depends on the information strength (sample size) and the uncertainty of gene-wise dispersion estimates. A larger sample size is accompanied by a lower shrinkage level, whereas greater deviation generally results in a higher shrinkage level. However, for certain genes with extremely high dispersion (more than two standard deviations above the fitted trend on the log scale), the original dispersion estimate is retained to avoid underestimation of true variability. For each gene, sample outliers are detected using Cook’s distance and handled to improve robustness. Whenever normalized counts are incorporated into estimation, they are generated using the default Relative Log Expression (RLE) normalization method.

DESeq, an earlier version preceding DESeq2, uses a method-of-moments strategy to estimate gene-specific dispersion and then fits a local regression to model the relationship between mean expression and dispersion across all genes. Unlike DESeq2, it does not apply empirical Bayes shrinkage, but instead adopts a more conservative approach by selecting the larger value between the gene-wise estimate and the fitted trend estimate to ensure robustness in variance estimation, especially for small sample sizes.

edgeR uses a similar NB GLM framework for dispersion estimation based on maximum likelihood, distinguishing common, gene-specific, and trended dispersion estimates. An empirical Bayes strategy is applied to shrink gene-specific estimates toward the common or trended dispersions automatically. edgeR offers two distinct statistical tests for differential expression: the likelihood ratio test (edgeR.lrt) and the quasi-likelihood F-test (edgeR.qlf). Whenever normalized data are required, the Trimmed Mean of M-values (TMM) method is used.

NBPSeq extends the traditional NB model by introducing an additional parameter (α) into the variance function, resulting in the Negative Binomial with Power (NBP) distribution. This extension allows for greater flexibility in modeling the relationship between dispersion and mean expression. For differential expression hypothesis testing, it adapts the Robinson and Smyth exact test for the NBP distribution, which is robust for small-sample RNA-seq data. Normalization was performed using TMM.

DSS, originally designed for single-nucleotide methylation data, also models RNA-seq data using the NB distribution. It assumes that gene-specific dispersion follows a log-normal prior distribution and estimates it using the method of moments combined with a penalized likelihood approach to borrow information across genes. For differential expression testing, DSS performs a Wald test based on stabilized, shrunk dispersion estimates. Default normalization follows a TMM/RLE-like log-ratio method to account for library size differences.

NOISeq is a non-parametric method that models or estimates noise directly from the dataset by comparing replicates within the same condition. It constructs a reference distribution of log-fold changes (M) and absolute differences (D) to determine how likely the observed differences between conditions are due to true differential expression rather than random noise. TMM normalization was applied.

ROTS employs a modified t-statistic to measure differential expression. Multiple datasets are generated by resampling, and the overlap degree (reproducibility) of gene rankings across resampled datasets is calculated. This overlap degree is maximized to estimate the t-statistic parameters. Normalization was performed using TMM, followed by voom transformation to prepare the data for linear modeling.

ABSSeq assumes that read count differences between two conditions follow a NB distribution. It uses the MAD to identify and account for outliers by robustly assessing variation across samples. The default normalization method is a quantile-based procedure designed to achieve better concordance with experimental quantification methods.

voom is a linear modeling approach for RNA-seq data that applies log transformation to convert count data into log-counts per million (logCPM), which are suitable for linear modeling. It estimates the mean–variance relationship of the data and generates a precision weight for each observation. These precision weights are subsequently incorporated into a linear model using the limma empirical Bayes framework for differential expression analysis. TMM normalization is applied to estimate effective library sizes prior to logCPM calculation.

The T.test is a classic parametric method suitable for differential expression analysis between two groups of samples. This method assumes that the data follow a normal distribution and is generally more appropriate for large sample sizes. We used logCPM-transformed values as input.

The Wilcoxon rank-sum test is a non-parametric method suitable for two-group differential expression analysis that does not rely on distributional assumptions. Normalization was performed using logCPM transformation prior to analysis.

## Performance evaluation metrics

### Area under the ROC curve (AUC) calculation

The Receiver Operating Characteristic (ROC) curve plots FPR on the x-axis and TPR on the y-axis, which visually measures a method’s effectiveness in distinguish different groups. The AUC has two major advantages: it does not depend on the selection of a specific threshold [32], and it is suitable for class-unbalanced datasets [33]. Therefore, AUC is often used to evaluate the performance of different differential analysis methods in detecting differentially expressed genes (DEGs). Since we introduce predefined DEGs through simulation, we can evaluate DE performance using a fully known set of DEGs and non-DEGs.

### Generation of FDR-TPR curves

To evaluate the performance of different DE methods, we calculated the TPR and the FDR, and plotted the FDR-TPR curve. TPR represents the proportion of all true positive classes that are correctly identified as significant, and FDR is the proportion of samples labeled as significant that are incorrectly identified as positives. First, the TPR and FDR under each threshold were calculated by sorting p-values and setting different ranks as significance thresholds, and then the FDR-TPR curve with FDR between 0 and 0.15 was drawn.

### Evaluation of false positive discoveries in real datasets

To evaluate false-positive control, we conducted testing under null model scenarios, performing DE analysis between samples from the same condition, where no true differences should exist. The proportion of falsely detected DEGs represents the FPR, assessing how well a method controls false positives. We used normal samples from TCGA-BRCA and SCLC datasets to perform 100 subsampling iterations, randomly dividing them into two groups of a specified sample size, and calculating the resulting false positive proportion.

### Concordance at the top (CAT) calculation

In order to evaluate the consistency and stability of the DE methods among data subsets, we used Concordance at the top (CAT) [34]. The specific steps are as follows: each real dataset is subsampled and randomly divided into two equal subsets with sample sizes of 3, 5, 10, 30, and 50, respectively, and subsampled 10 times to reduce random errors. Then, we compared the top 10% gene lists from each method by sorting genes from smallest to largest p-value. Similarly, the stability of a method is also assessed by comparing the results of the same method in different comparisons.

### Correlation of pathway enrichment results

To assess functional analysis consistency, we computed Spearman’s correlation coefficients between pathway enrichment results across methods. Pathways were selected if their adjusted p-values were < 0.05 in any method. Lastly, we calculated the Spearman correlation coefficient for the normalized enrichment score (NES) of each method to assess the similarity across methods.

### Synthetic and real datasets

We generated simulation expression matrices from three diverse public datasets, including cell line comparisons, tissue comparisons, and cancer datasets. E-ENAD-34, a published dataset, compares the gene expression of mature neutrophils in smokers and non-smokers, with 34 and 60 replicates for each condition. This dataset was retrieved from the EMBL-EBI database via the ExpressionAtlas package [35] (version 1.30.0). GSE150910 [36], a lung tissue RNA-seq dataset from NCBI GEO, covering patients with idiopathic pulmonary fibrosis (IPF) and healthy controls, with 103 samples in each group. The TCGA-LUSC dataset [37,38] is from the Cancer Genome Atlas (TCGA) project, comprising 497 lung squamous cell carcinoma (LUSC) tumor samples and 51 adjacent normal tissue samples. TCGA-LUSC is thus representative of the complexity and heterogeneity in cancer research. We downloaded this dataset using the TCGAbiolinks package [39–41] (version 2.30.4).

For real data analysis, we used the TCGA-BRCA dataset. TCGA-BRCA was chosen for the larger sample size required by this study (at least 100 samples for each condition). Count data were also downloaded from the TCGAbiolinks package. To evaluate generalization in an independent tumor transcriptome dataset, we additionally used the SCLC dataset reported by Liu et al. [42], containing 107 tumor and 107 normal samples. In the process of data preprocessing, the Euclidean distance matrix of the Normal and Cancer group samples was calculated after t-SNE dimensionality reduction. Next, potential outliers were identified by calculating the average Euclidean distance between each sample and all others. Samples exhibiting distances exceeding the threshold—defined as the 75th percentile plus twice the interquartile range (IQR) —were removed as outliers.

### NB and Poisson simulation framework

NB and Poisson simulations were generated to evaluate performance under different count-generating assumptions and perturbations. Quality control was first performed at both the sample and gene levels. For samples, outlier samples were identified via t-SNE visualization (perplexity = 30, iterations = 1,000) using the Rtsne package, with samples deviating from the main cluster removed. For genes, those with a mean expression count below seven across samples were excluded. Dispersion parameters and gene-wise means were estimated separately for case and control groups using edgeR. To simulate biologically relevant differences, we selected 1,500 upregulated and 1,500 downregulated genes, multiplying their means by the respective fold changes. Using these adjusted parameters, we generated expression matrices with 10,000 genes and sample sizes of *n* = 3, 5, 10, 30, or 50 per group, repeating the process 100 times. This strategy was adapted from prior work by Baik et al. [43].

### NB simulations

We used simulated data based on the NB distribution. Specifically, for the count $Y_{ig}$ of gene g in sample i, the probability distribution is given by:


$$\begin{array}{c} Y_{ig}\sim NB(\mu_{ig},\phi_{g}) \end{array}$$

where $\mu_{ig}=s_{i}\times p_{g}$ is the mean parameter (si is the library size factor for sample i, and pg is the expression proportion of gene g), and ϕg is the gene-specific dispersion parameter. Maximum likelihood estimates of the parameters for both the treatment and control groups were obtained using the edgeR package (version 3.42.0).

### Poisson simulations

Poisson simulations used the same mean parameter as the NB simulations but did not include a gene-specific dispersion parameter:


$$\begin{array}{c} Y_{ig}\sim Poisson(\lambda_{ig}) \end{array}$$

where $\lambda_{ig}=s_{i}\times p_{g}$ is the expected count. Gene-specific average expression levels were estimated separately for the case and control groups and used as Poisson rate parameters. Poisson matrices were generated using the same DE proportion, fold changes, sample sizes, and numbers of replicates as the NB simulations.

### Outlier-perturbation simulations

Outlier perturbations were introduced into both standard NB and Poisson simulations to assess robustness to extreme expression values. Outlier proportions were set at 0.1%, 0.5%, 1%, 2%, and 5%, with 100 replicates per sample size and outlier level. This approach evaluated model robustness against data variability and outliers.

### Random-noise simulations

Multiplicative random noise was introduced into both standard NB and Poisson simulations to model increased gene-level random variation. A relatively low-variation dataset was used as the parameter source. Before count generation, each gene-expression mean was multiplied by a random factor drawn from 1−r to 1 + *r*, where the noise level *r* was 0, 0.2, 0.4, 0.6, or 0.8. For example, when *r* = 0.2, the mean was multiplied by a random value between 0.8 and 1.2. One hundred replicates were generated for every distribution, sample size, and noise level. The slope of AUC against noise level quantified noise sensitivity, with a smaller absolute slope indicating stronger noise robustness.

### Visualization of integrated multidimensional performance scores

To summarize performance across multiple dimensions in one visualization, each metric was converted into a normalized performance score and displayed as a bubble plot. Each bubble represented the relative performance of one method at a specific sample size and evaluation dimension; both bubble size and color mapped to the normalized score.

Raw performance values were first calculated for every dimension. Mean AUC represented overall discrimination in standard simulations. Mean TPR in the low-FDR region represented low-FDR sensitivity. Outlier and noise robustness were quantified by the absolute slopes of AUC against perturbation level. Observed FPR under null-model conditions represented false-positive control. CAT of the top 10% gene lists represented stability across data subsets.

All evaluation metrics were oriented such that larger values indicated better performance. AUC, mean low-FDR TPR, and CAT were used directly without transformation. Outlier robustness and noise robustness were quantified using the negative absolute value of the perturbation slope, i.e., −|β|, where β denotes the estimated performance–perturbation regression coefficient. FPR control was evaluated using the negative observed FPR to ensure consistency in directionality across metrics.

Within each evaluation dimension, all direction-aligned metrics were rescaled using min–max normalization:


$$\begin{array}{c} score=\frac{x-\min(x)}{\max(x)-\min(x)}. \end{array}$$

If all values within a dimension were identical, all scores were set to one. The resulting scores represent relative performance within each evaluation dimension and should not be interpreted as directly comparable absolute performance across dimensions.

### Ethics statement

This study was based on publicly available datasets and did not involve the recruitment of human participants. Therefore, ethical approval was not applicable.

## Results

### Benchmarking framework for DE methods

**Fig 1:**
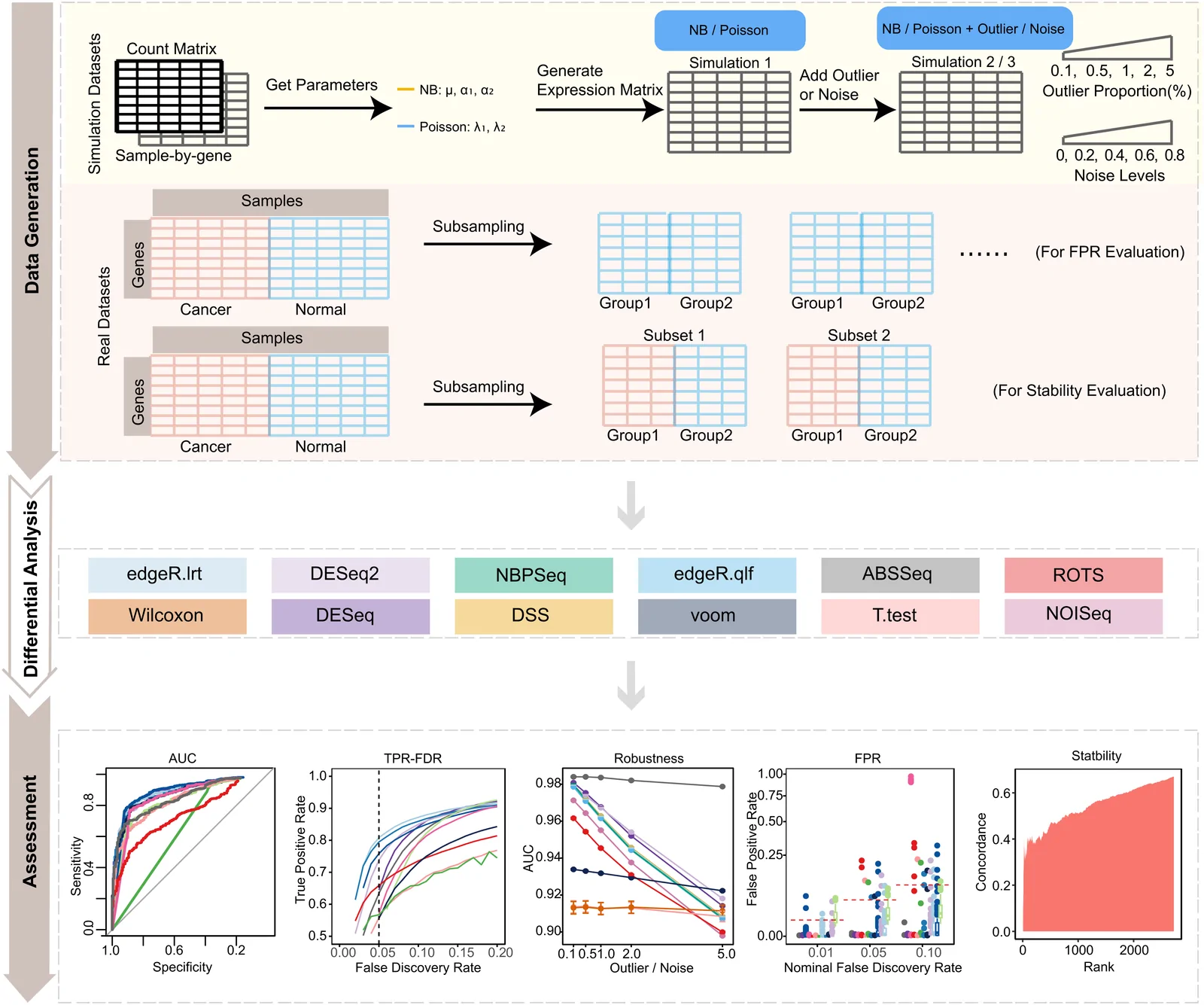
Benchmarking framework for DE methods. This study systematically evaluates 12 DE methods using both simulated and real data. Three stages are involved: data generation, differential analysis, and assessment. We designed six simulation scenarios: (1) standard NB distribution data, (2) NB data with outliers, (3) NB data with added noise, (4) standard Poisson-based data, (5) Poisson data with outliers, and (6) Poisson data with added noise. The real data were subsampled for FPR evaluation and stability evaluation. The performance of the DE methods is evaluated based on six properties: (1) AUC; (2) TPR curve at low FDR thresholds; (3-4) robustness under outlier or noise conditions; (5) false-positive control ability; and (6) cross-dataset stability.

Fig 1 summarizes our benchmarking framework for differential expression analysis. We evaluated 12 DE methods, including edgeR implemented with two commonly used pipelines, using both simulated and real RNA-seq count data. The workflow has three stages: data generation, differential analysis, and performance assessment. Six simulation scenarios were constructed to represent increasing departures from ideal assumptions: standard NB counts, NB counts with injected outliers, NB counts with added noise, standard Poisson counts, Poisson counts with injected outliers, and Poisson counts with added noise. These settings allow us to measure baseline accuracy and robustness under challenging conditions. For real-data evaluation, we used subsampling strategies to assess false positive behavior and result stability. Method performance was quantified by six properties: (1) area under the ROC curve (AUC) for overall discrimination; (2) true positive rate (TPR) at low false discovery rate (FDR) thresholds to reflect practical operating regions; (3–4) robustness to outliers and noise; (5) false positive rate (FPR) control on real data; and (6) cross-dataset stability to evaluate reproducibility across datasets.

### NB simulation results

The absence of biological ground truth makes it difficult to use real datasets to evaluate different analysis methods. It is widely accepted that the NB distribution, with its additional dispersion parameter, better captures the overdispersion characteristic of RNA-seq data. Therefore, we first constructed simulated datasets based on the NB distribution to compare the statistical properties of different differential analysis methods. The additional dispersion parameter of the NB distribution enables it to capture the overdispersion commonly observed in RNA-seq data. Simulations were performed using three RNA-seq source datasets: E-ENAD-34, GSE150910, and LUSC. The performance of these methods was assessed using two metrics: AUC and the TPR curve at low FDR thresholds.

**Fig 2:**
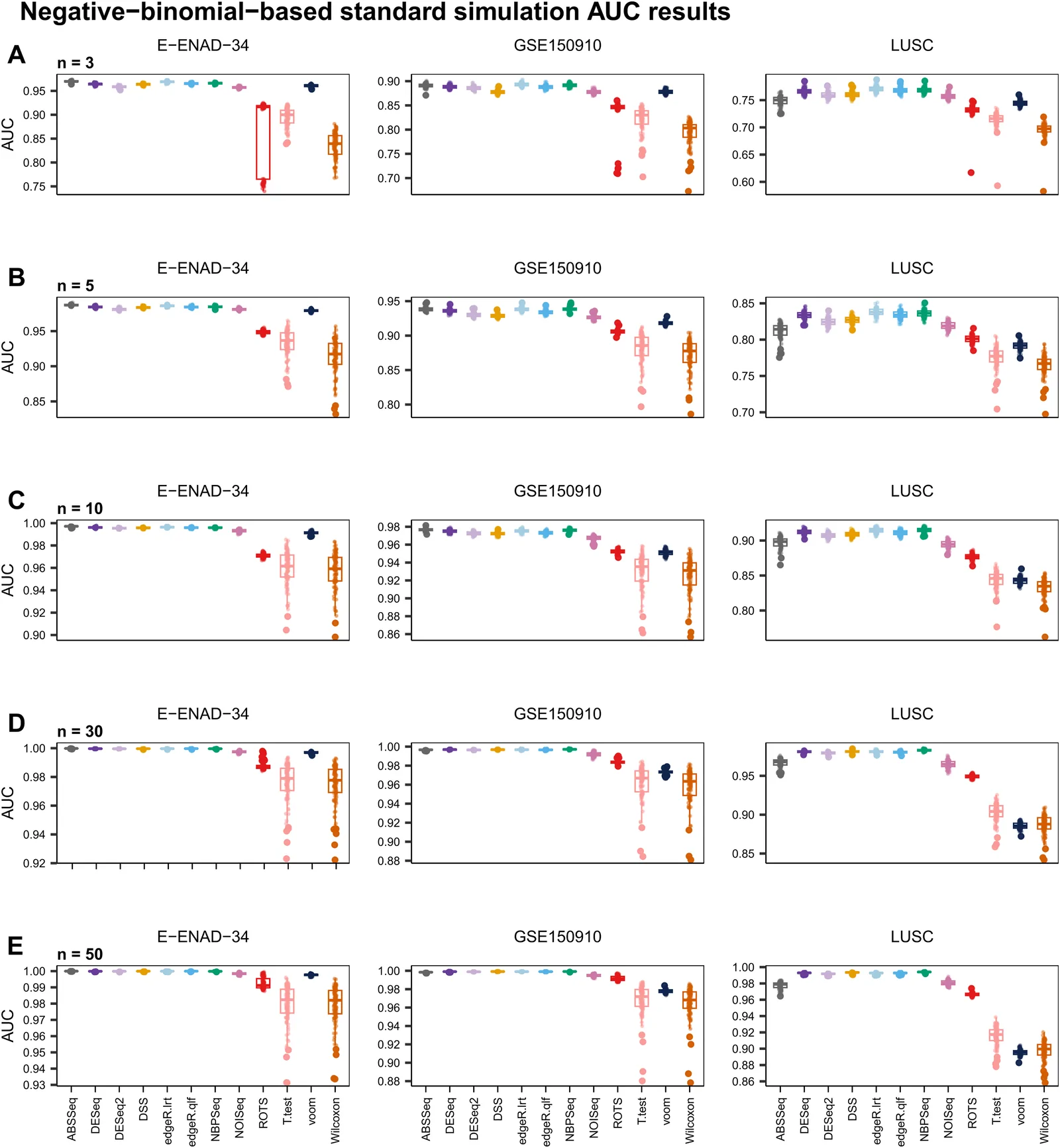
AUC results based on NB distribution simulations of different sample sizes. A) AUC of DE methods in simulated datasets with a sample size of *n* = 3. The boxplots summarize AUC values obtained from 100 independent simulations for each dataset. The three panels correspond to simulations based on three real datasets. B) Same as A), but with a sample size of *n* = 5. C) Same as A), but with a sample size of *n* = 10. D) Same as A), but with a sample size of *n* = 30. E) Same as A), but with a sample size of *n* = 50.

**Fig 3:**
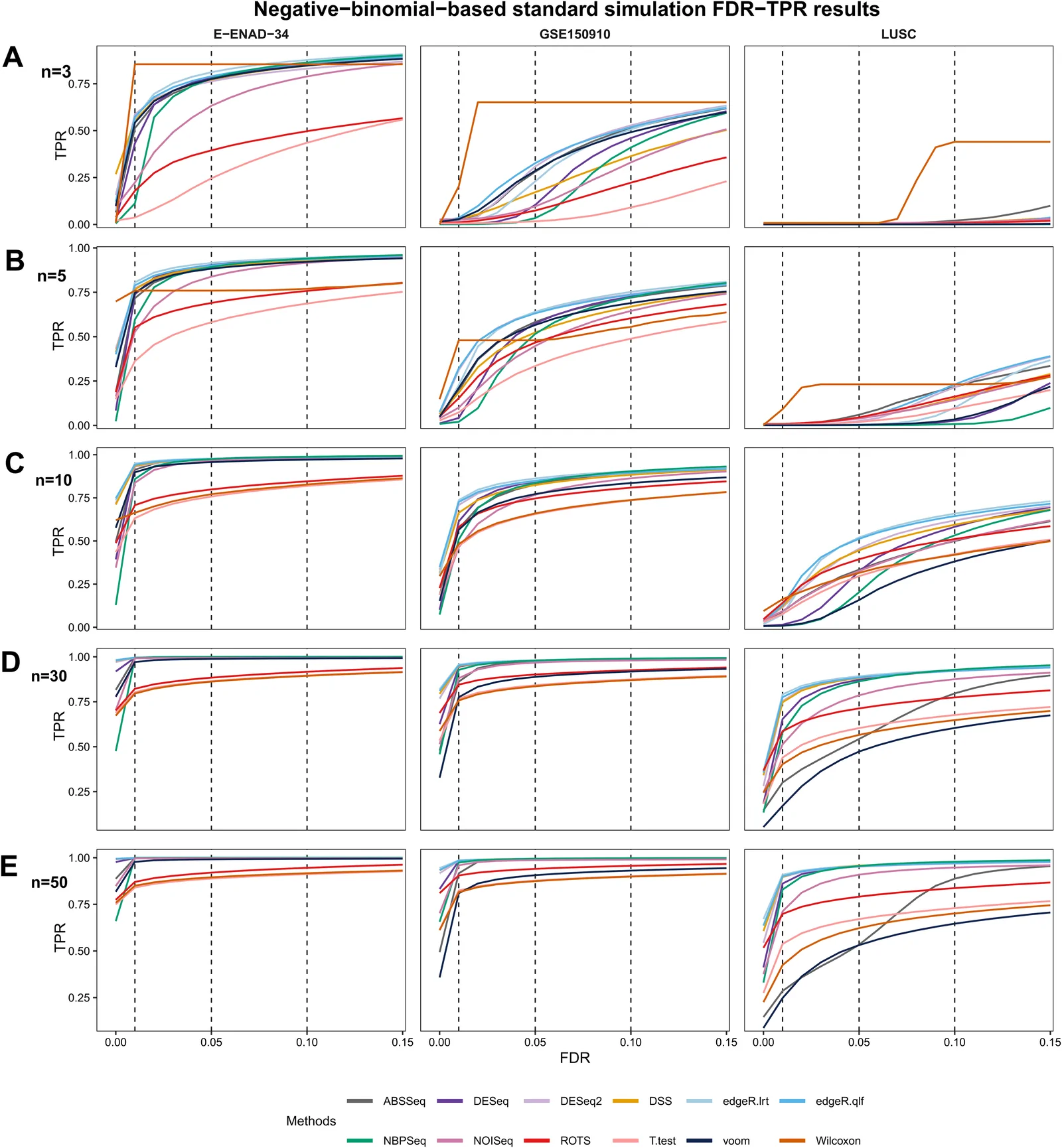
FDR-TPR results based on NB distribution simulations of different sample sizes. A) FDR-TPR curves of DE methods in simulated datasets with a sample size of *n* = 3. The three dashed lines indicate the target FDR levels of 0.01, 0.05, and 0.1. Each curve represents the average true positive rate (TPR) at a given FDR (rounded to two decimal places), calculated across 100 independent simulations. Except for the voom method, other methods based on the NB distribution demonstrate better overall performance. B) Same as A), but with a sample size of *n* = 5. C) Same as A), but with a sample size of *n* = 10. D) Same as A), but with a sample size of *n* = 30. E) Same as A), but with a sample size of *n* = 50.

In the NB simulations, edgeR.lrt, NBPSeq, edgeR.qlf, DESeq2, and DSS generally exhibit better performance(Fig 2). However, the overall AUC values obtained from the LUSC dataset are lower than those from the other two datasets, likely due to the greater biological variability present in LUSC, which poses a more substantial challenge for all methods (S1 Fig in S1 File). Notably, voom and ABSSeq perform significantly worse on the LUSC dataset compared to other methods, suggesting that their stability across datasets is relatively poor. When the sample size is 10, edgeR.qlf, edgeR.lrt, DESeq2, and DSS display superior FDR–TPR curves, indicating stronger sensitivity in detecting differentially expressed genes at low false discovery rates (Fig 3A-E).

Overall, in terms of AUC, edgeR.lrt outperforms edgeR.qlf, suggesting better general validity. However, in terms of FDR–TPR performance, edgeR.qlf has an advantage, implying better performance in identifying DEGs under stricter FDR constraints. Although NBPSeq achieves a high AUC even when the sample size is below 10, its FDR–TPR performance is less impressive (Figs 2, Fig 3), indicating that while it has strong overall validity, it may not be the optimal choice when dealing with small sample sizes and requiring strict false-positive control.

### Poisson simulation results

To determine whether method performance depended on a specific count-data-generating distribution, we additionally performed Poisson simulations. The Poisson distribution represents a more idealized count-generating process without explicit overdispersion. The source datasets and evaluation metrics were the same as those used for the NB simulations.

**Fig 4:**
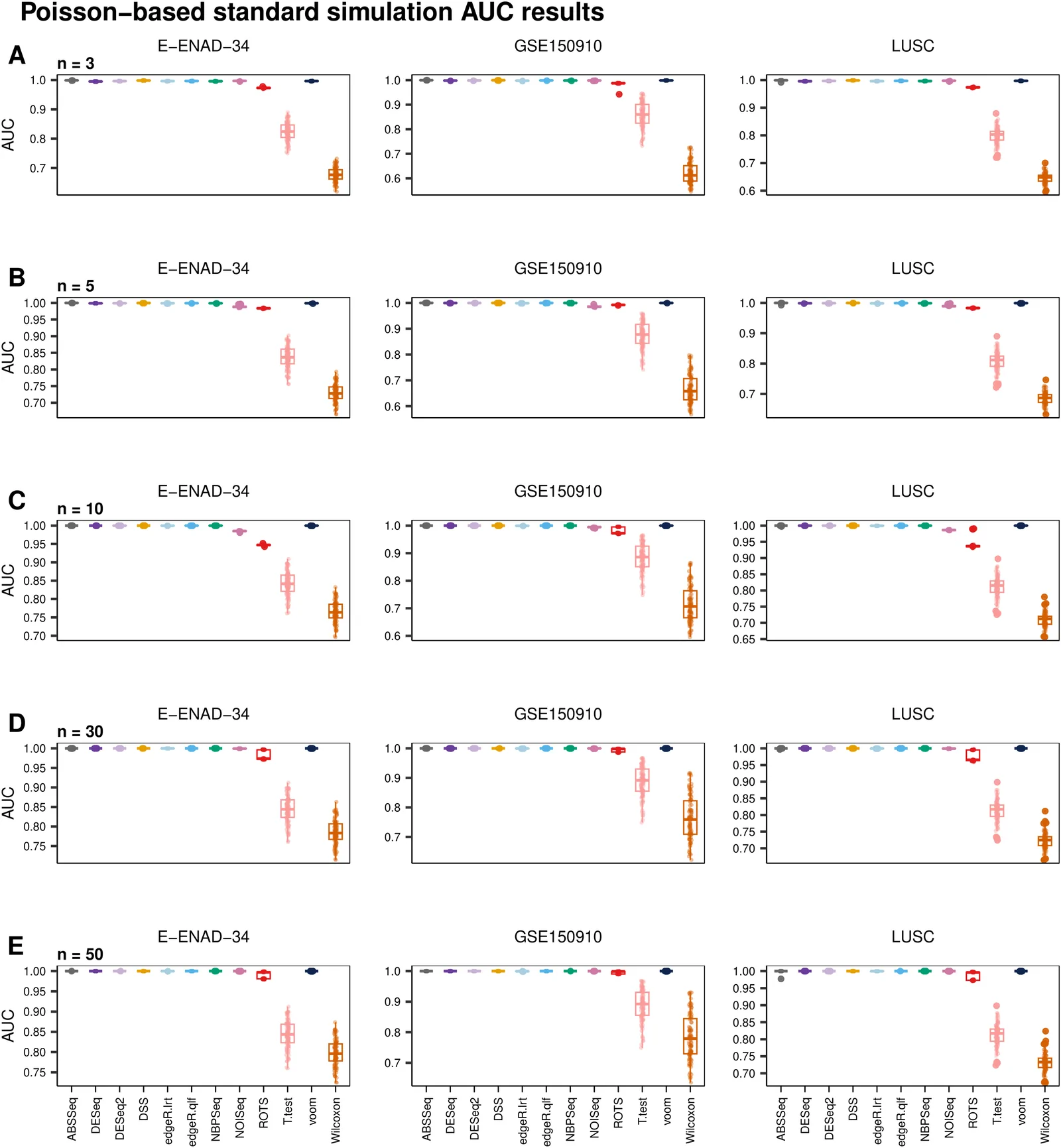
AUC results based on Poisson-based standard simulations of different sample sizes. A) AUC of DE methods in simulated datasets with a sample size of *n* = 3. The boxplots summarize AUC values obtained from 100 independent simulations for each dataset. The three panels correspond to simulations based on three real datasets. B) Same as A), but with a sample size of *n* = 5. C) Same as A), but with a sample size of *n* = 10. D) Same as A), but with a sample size of *n* = 30. E) Same as A), but with a sample size of *n* = 50.

In the Poisson simulations, ABSSeq, DESeq, DESeq2, DSS, edgeR.lrt, edgeR.qlf, NBPSeq, NOISeq, and voom generally performed well, whereas ROTS showed slightly weaker performance (Fig 4). Because the Poisson distribution does not include an additional dispersion parameter, differences among the source datasets were smaller than those observed in the NB simulations. The T.test and Wilcoxon test performed substantially worse than the other methods across all source datasets. Besides, their performance improved only modestly with increasing sample size, unlike the count-based methods.

**Fig 5:**
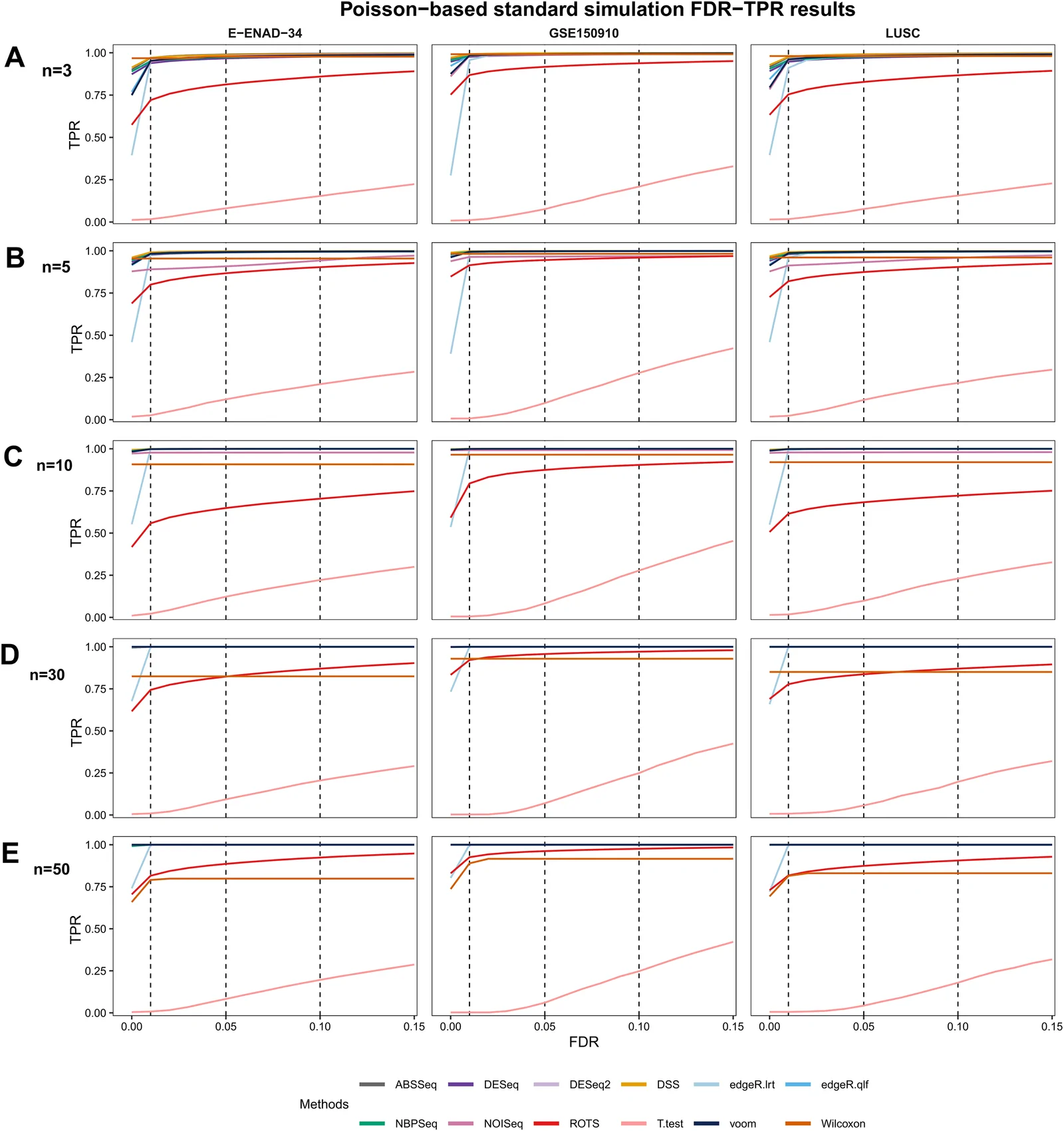
FDR-TPR results based on Poisson-based standard simulations of different sample sizes. A) FDR-TPR curves of DE methods in simulated datasets with a sample size of *n* = 3. The three dashed lines indicate the target FDR levels of 0.01, 0.05, and 0.1. Each curve represents the average true positive rate (TPR) at a given FDR (rounded to two decimal places), calculated across 100 independent simulations. Most count-based methods achieved higher TPRs in the low-FDR region, whereas the T.test, ROTS, and Wilcoxon test showed weaker low-FDR sensitivity. B) Same as A), but with a sample size of *n* = 5. C) Same as A), but with a sample size of *n* = 10. D) Same as A), but with a sample size of *n* = 30. E) Same as A), but with a sample size of *n* = 50.

Most DE methods, with the exception of the T.test, ROTS, and Wilcoxon test, achieved better FDR–TPR curves in the Poisson simulations. The weaker low-FDR performance of the T.test, ROTS, and Wilcoxon test, particularly at moderate and large sample sizes, may reflect suboptimal p-value ranking or FDR calibration for discrete count data rather than simply insufficient statistical power (Fig 5).

### Outlier simulations based on the NB distribution

In real RNA-seq data, the expression levels of certain genes may exhibit extremely high or low values in some samples. These extreme values are commonly referred to as outliers. Outliers may arise from sequencing errors, technical noise, or biological factors, such as unusually high expression of a gene in specific samples. If not properly handled, outliers in actual data can lead to false positives or false negatives. To evaluate the performance of differential expression analysis methods under such conditions and to better understand their robustness in real-world scenarios, we introduced random outliers into simulations based on the NB distribution.

**Fig 6:**
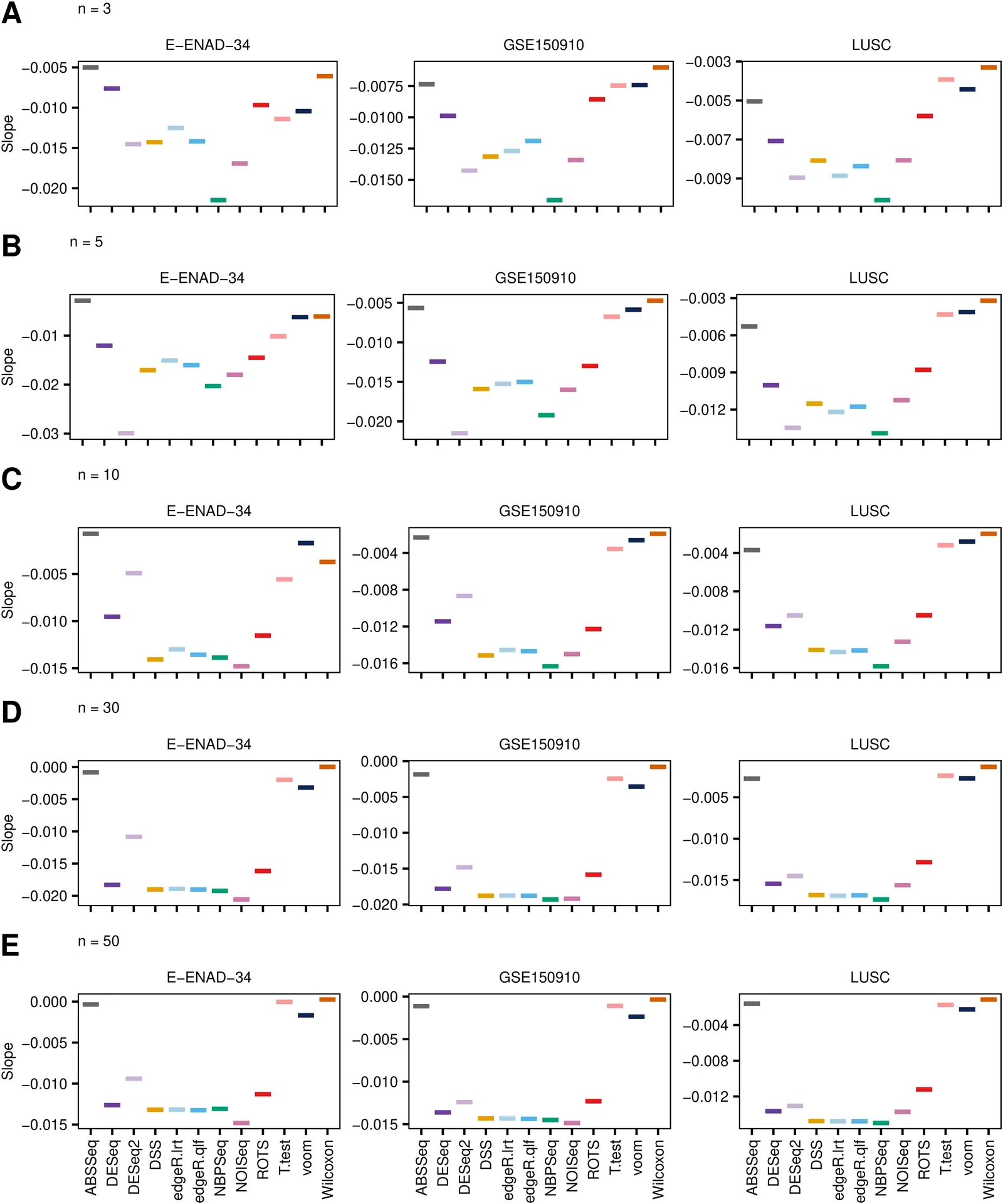
Robustness of DE methods in NB simulations with outliers of different sample sizes. A) Systematic analysis of AUC changes for each method under varying outlier proportions in three types of simulated datasets (sample size = 3). Outlier levels were set to 0.1%, 0.5%, 1%, 2%, and 5%. For each method, the slope of the AUC change was calculated to quantify robustness. A smaller slope indicates greater robustness (i.e., lower sensitivity to outliers). B) Same as A), but with a sample size of *n* = 5. C) Same as A), but with a sample size of *n* = 10. D) Same as A), but with a sample size of *n* = 30. E) Same as A), but with a sample size of *n* = 50.

S2 Fig in S1 File presents trend plots of AUC values as the proportion of outliers increases. To more clearly illustrate the sensitivity of each method to outliers, we calculated the slope of each line segment in S2 Fig in S1 File and visualized them as box plots (Fig 6). The slopes of ABSSeq, the T.test, and the Wilcoxon test were consistently closer to zero compared to those of other methods, indicating that their performance was less affected by increasing outlier proportions and thus more robust. This suggests that deviations from the NB distribution caused by outliers can impair the performance of other methods that rely on NB assumptions. However, ABSSeq appears to mitigate this effect effectively by leveraging the median absolute deviation (MAD) to detect and adjust for outliers.

Moreover, as the sample size increases, the slopes of all methods decrease, indicating improved robustness across the board. In other words, larger sample sizes enhance the stability and tolerance of differential expression analysis methods against data irregularities (Fig 6).

### Outlier simulations based on the Poisson distribution

To assess robustness under a different data-generating distribution, random outliers were also introduced into the Poisson simulations.

**Fig 7:**
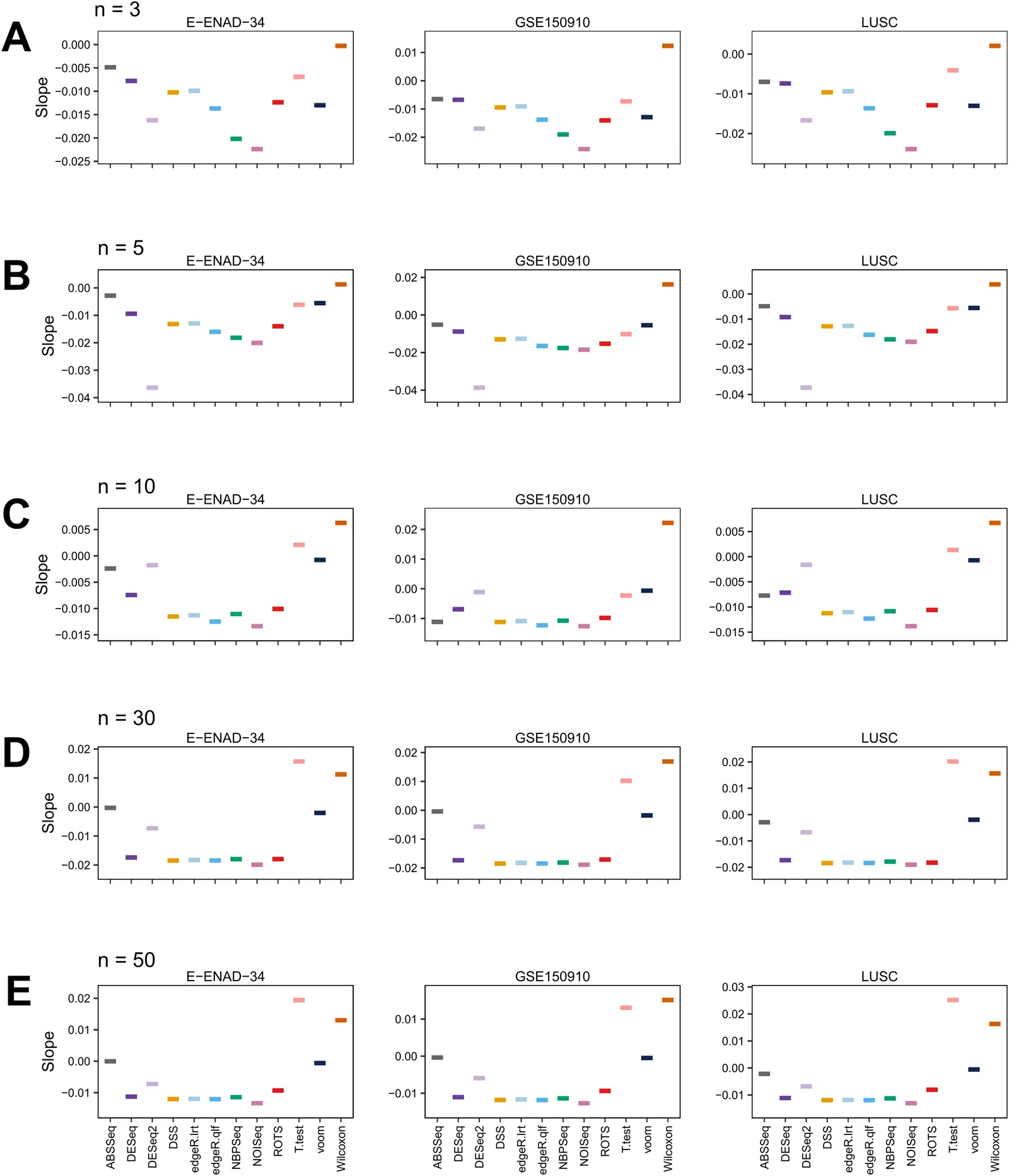
Robustness of DE methods to outliers in Poisson simulations of different sample sizes. A) Systematic analysis of AUC changes for each method under varying outlier proportions in three types of simulated datasets (sample size = 3). Outlier levels were set to 0.1%, 0.5%, 1%, 2%, and 5%. For each method, the slope of the AUC change was calculated to quantify robustness. A smaller slope indicates greater robustness (i.e., lower sensitivity to outliers). B) Same as A), but with a sample size of *n* = 5. C) Same as A), but with a sample size of *n* = 10. D) Same as A), but with a sample size of *n* = 30. E) Same as A), but with a sample size of *n* = 50.

As the outlier proportion increased in the Poisson simulations, the AUC trends indicated that the T.test and Wilcoxon test were relatively insensitive to outlier contamination in some settings. This pattern was further supported by their slopes, which were generally closer to zero than those of most other methods (S3 Fig in S1 File, Fig 7), consistent with their behavior in the NB simulations.

Consistent with the NB outlier simulations, larger sample sizes improved the stability of DE methods under data irregularities, as the slopes of all methods moved closer to zero (Fig 7A-E).

### Noise simulations based on the NB distribution

**Fig 8:**
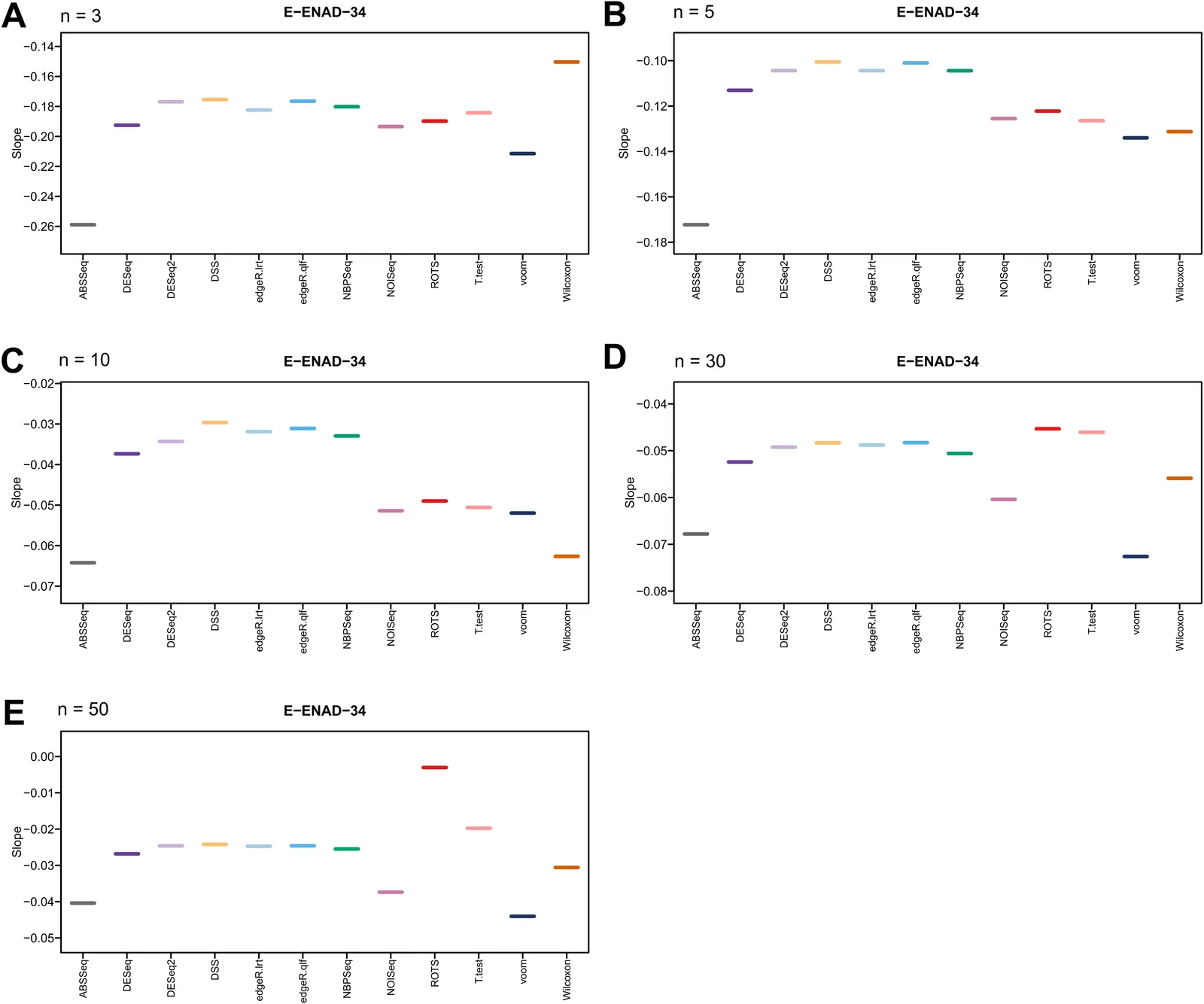
Robustness of DE methods to noise in NB simulations of different sample sizes. A) Slopes of AUC changes for each method under different levels of noise in simulations with a sample size of *n* = 3. Noise levels were set to 0.2, 0.4, 0.6, and 0.8. The slope of the AUC decline was calculated to quantify robustness. A smaller slope indicates better robustness, i.e., lower sensitivity to noise. B) Same as A), but with a sample size of *n* = 5. C) Same as A), but with a sample size of *n* = 10. D) Same as A), but with a sample size of *n* = 30. E) Same as A), but with a sample size of *n* = 50.

A key difference between datasets lies in whether genes exhibit greater variability (S1 Fig in S1 File). To simulate scenarios with increased gene-level variance, we selected a dataset with relatively low variability and introduced different levels of noise into the simulated data based on the NB distribution. S4 Fig in S1 File and Fig 8 illustrate the trend of AUC changes and the corresponding slopes, respectively, as noise levels increase. The results show that voom and ABSSeq were most affected by noise, indicating lower robustness to such variability. Voom relies on modeling the mean-variance relationship, which may adapt poorly when dispersion increases, whereas the robust strategy of ABSSeq may be better suited to isolated outliers than to broad random noise.

All methods showed negative AUC slopes as noise increased, indicating that random noise caused more extensive performance loss than outlier perturbation (Fig 8A-E). At *n* = 3 per group, Wilcoxon showed the smallest AUC decline in this specific noise-slope analysis. At a moderate sample size of *n* = 10, edgeR.lrt and edgeR.qlf were less affected by noise. ROTS, which learns parameters through resampling, showed smaller AUC declines at larger sample sizes of 30 or more.

### Noise simulations based on the Poisson distribution

**Fig 9:**
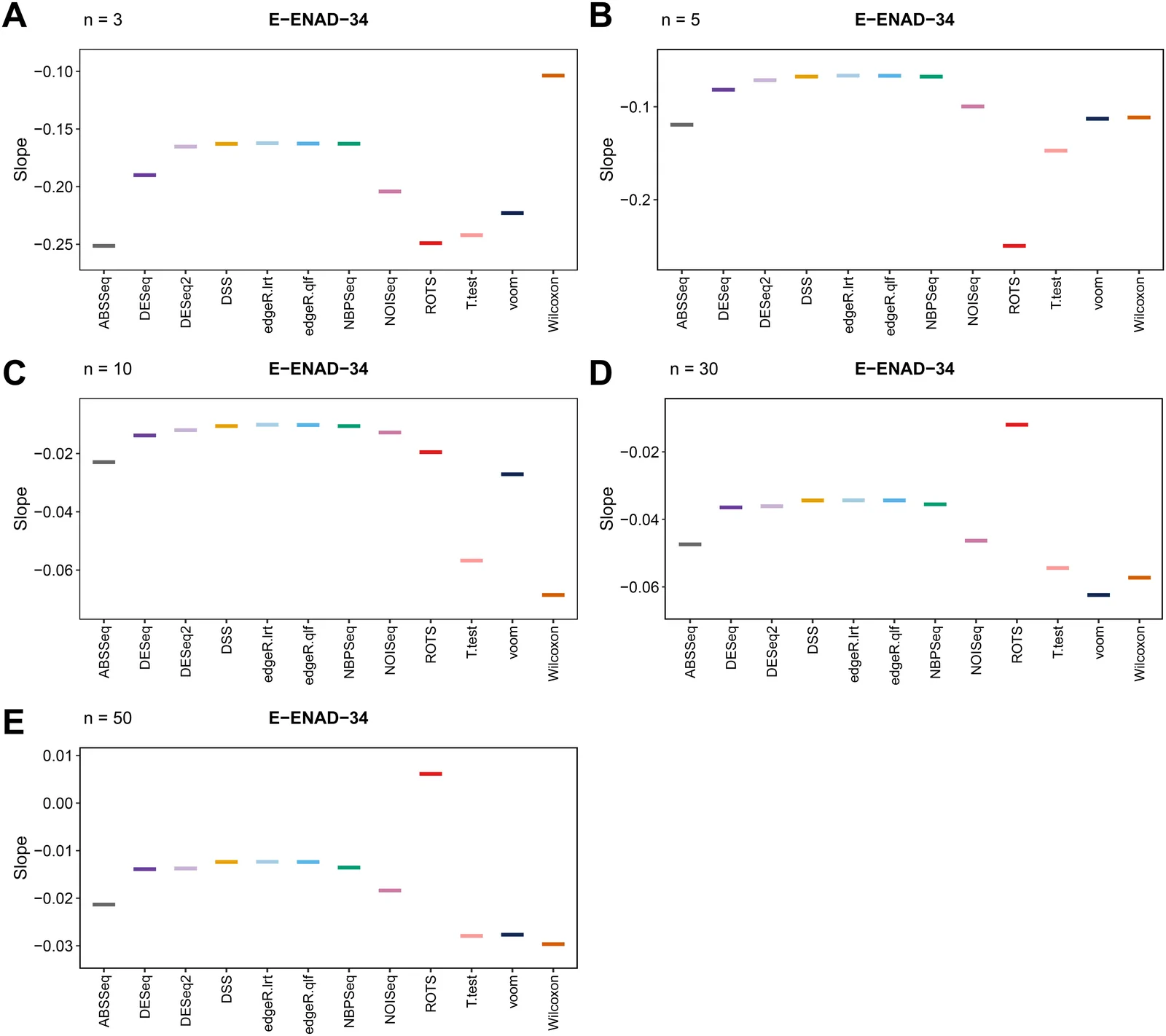
Robustness of DE methods to noise in Poisson simulations of different sample sizes. A) Slopes of AUC changes for each method under different levels of noise in simulations with a sample size of *n* = 3. Noise levels were set to 0.2, 0.4, 0.6, and 0.8. The slope of the AUC decline was calculated to quantify robustness. A smaller slope indicates better robustness, i.e., lower sensitivity to noise. B) Same as A), but with a sample size of *n* = 5. C) Same as A), but with a sample size of *n* = 10. D) Same as A), but with a sample size of *n* = 30. E) Same as A), but with a sample size of *n* = 50.

To assess whether noise robustness depended on the data-generating distribution, different noise levels were introduced into Poisson simulations generated using the same source dataset as the NB noise analysis. In the Poisson noise simulations, increasing noise similarly reduced AUC for all methods, with the T.test, Wilcoxon test, voom, and ABSSeq showing the strongest performance declines (S5 Fig in S1 File, Fig 9). The T.test is sensitive to variance inflation, whereas widespread noise can disrupt the between-group expression ranks on which the Wilcoxon test relies.

As in the NB noise simulations, AUC decreased with increasing noise for all methods (Fig 9). At a small sample size of *n* = 3, Wilcoxon showed the smallest AUC decline in this specific noise-slope analysis. At a moderate sample size of *n* = 10, DESeq, DESeq2, DSS, edgeR.lrt, edgeR.qlf, and NBPSeq were less affected by noise. ROTS showed the strongest noise robustness at n≥30 per group, consistent with the smaller AUC declines observed in the NB noise simulations.

### Concordance within and among differential expression methods

We evaluated the stability of DE methods across subsets of real datasets. Each pair of subsets was obtained by downsampling samples under two conditions from the same expression matrix. DE analysis was then performed on each subset, and genes were ranked in ascending order according to their p-values. Concordance was calculated as the proportion of genes that overlapped within the top 10% of the ranked gene lists. This process was repeated 10 times. Intuitively, a stable DE method should select similar gene sets across different data.

**Fig 10:**
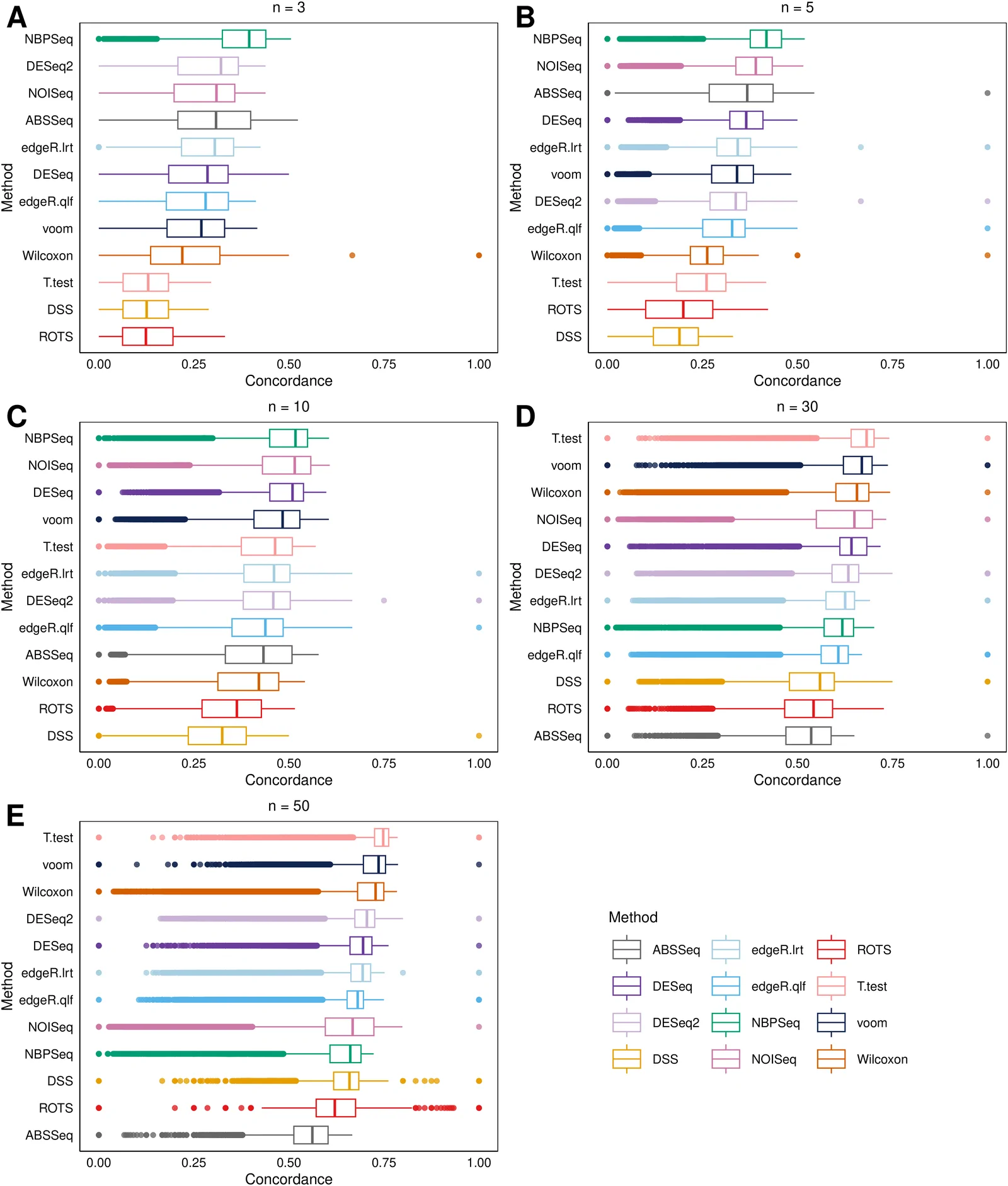
Stability of DE methods in TCGA-BRCA of different sample sizes. A) Boxplot of Concordance at the Top (CAT) across 10 iterations with a sample size of *n* = 3. B) Same as A), but with a sample size of *n* = 5. C) Same as A), but with a sample size of *n* = 10. D) Same as A), but with a sample size of *n* = 30. E) Same as A), but with a sample size of *n* = 50.

**Fig 11:**
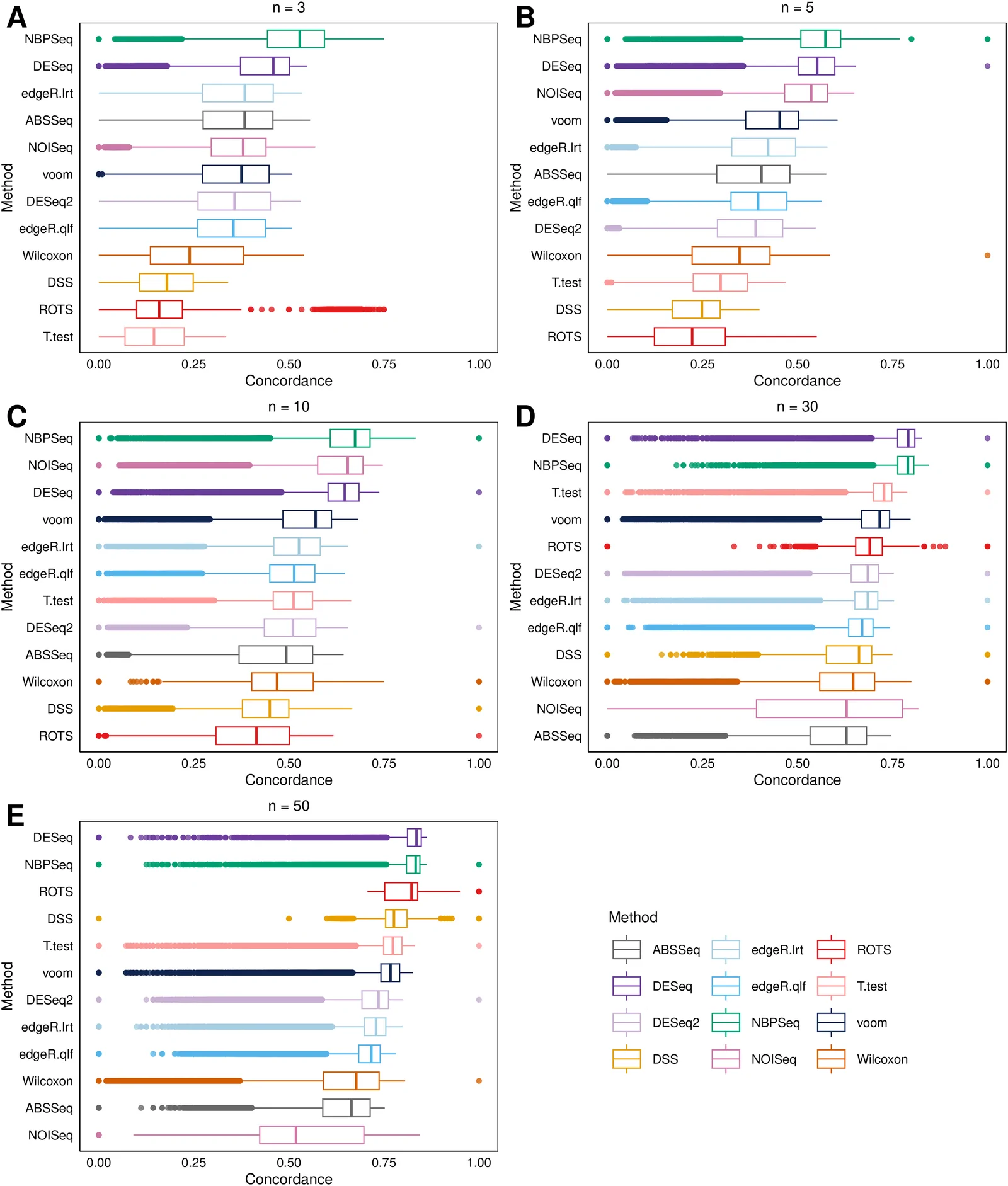
Stability of DE methods in SCLC dataset of different sample sizes. A) Boxplot of Concordance at the Top (CAT) across 10 iterations with a sample size of *n* = 3. B) Same as A), but with a sample size of *n* = 5. C) Same as A), but with a sample size of *n* = 10. D) Same as A), but with a sample size of *n* = 30. E) Same as A), but with a sample size of *n* = 50.

We found that when n≤10, NBPSeq consistently exhibited the highest concordance, followed by NOISeq, DESeq2, ABSSeq, and edgeR.lrt (Fig 10A-B). In BRCA, as the sample size increased, the median concordance of NBPSeq improved from approximately 0.4 to around 0.65 (Fig 10A-E), indicating enhanced stability with larger sample sizes. In contrast, DSS showed the lowest concordance when the sample size was below 10 (Fig 10A-B), suggesting that it is more sensitive to noise and outliers in small-sample scenarios. However, when n≥10, the T.test and voom demonstrated higher concordance (Fig 10C-E), indicating that they yield more stable and consistent results with sufficient sample sizes of n≤10. In SCLC, NBPSeq achieved consistently high concordance (Fig 11A-E). As sample sizes increase to 10 or more, the relative ranking of ROTS improved, whereas the rankings of ABSSeq and NOISeq declined because their concordance improved less. Thus, although increasing sample size tended to improve consistency, its effect differed across methods and datasets.

Inspired by the ribbon plots from Calgaro et al. [44], we further analyzed the consistency among different DE methods. We observed that edgeR.lrt, edgeR.qlf, and DESeq2 exhibited high similarity (S6-S10 Figs in S1 File), and their results remained relatively consistent across different sample sizes. Interestingly, when n≤10, voom clustered together with edgeR.lrt, edgeR.qlf, and DESeq2, indicating a high level of concordance among these methods. However, when the sample size reached 30 or above, voom began clustering with the T.test and Wilcoxon, forming a new group (S6-S15 Figs in S1 File). This may be attributed to voom’s use of a linear modeling approach, which aligns its results more closely with standard T.test as the sample size increases.

Finally, we investigated the similarity of downstream pathway enrichment results across DE methods. Once again, edgeR.lrt, edgeR.qlf, and DESeq2 demonstrated the strongest correlation. Overall, the clustering patterns in enrichment analysis resembled those observed in differential expression analysis (S6-S15 Figs in S1 File), indicating that the choice of DE method significantly impacts downstream functional interpretation. For example, at a sample size of *n* = 3, edgeR.lrt, edgeR.qlf, DESeq2, and DESeq consistently clustered together in both differential expression and enrichment analyses (S6 Fig, S11 Fig in S1 File). At a sample size of n = 50, edgeR and DESeq2 clustered with Wilcoxon and the T.test, while DESeq and NBPSeq showed a similar pattern (S10 Fig, S15 Fig in S1 File).

### False-positive rates of differential expression methods in real data

In differential expression analysis, the FPR is a key metric for evaluating method performance. FPR refers to the proportion of genes incorrectly identified as differentially expressed. To assess the FPR of each method, we randomly assigned labels to normal samples from the BRCA and SCLC datasets, and repeated this process 100 times.

**Fig 12:**
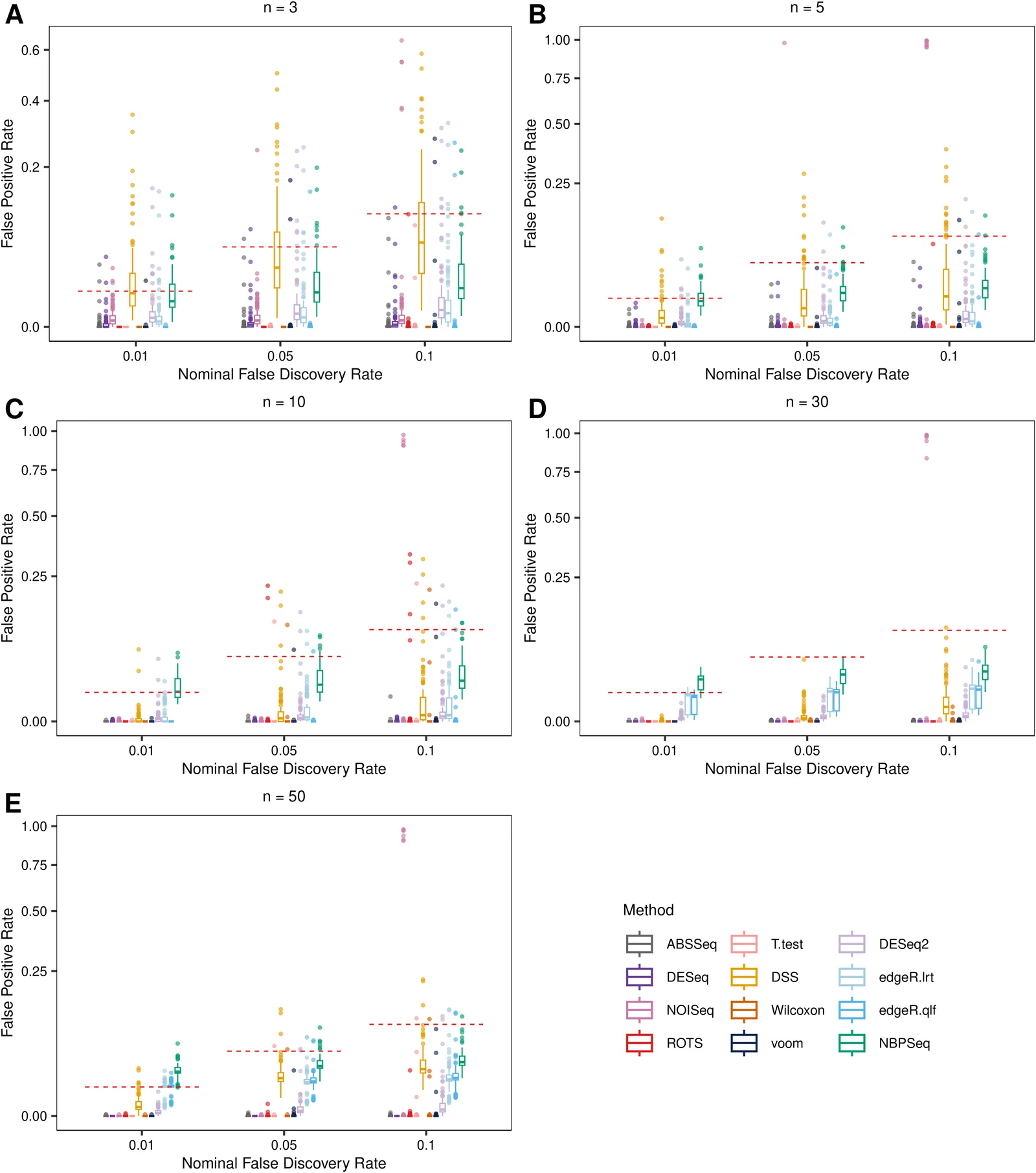
False positive rate (FPR) of DE methods in TCGA-BRCA of different sample sizes. A) FPR results with a sample size of *n* = 3. Each plot shows the actual FPR when the nominal FDR is set to 0.01, 0.05, and 0.1. The red dashed lines indicate the corresponding expected FPR values. B) Same as A), but with a sample size of *n* = 5. C) Same as A), but with a sample size of *n* = 10. D) Same as A), but with a sample size of *n* = 30. E) Same as A), but with a sample size of *n* = 50.

**Fig 13:**
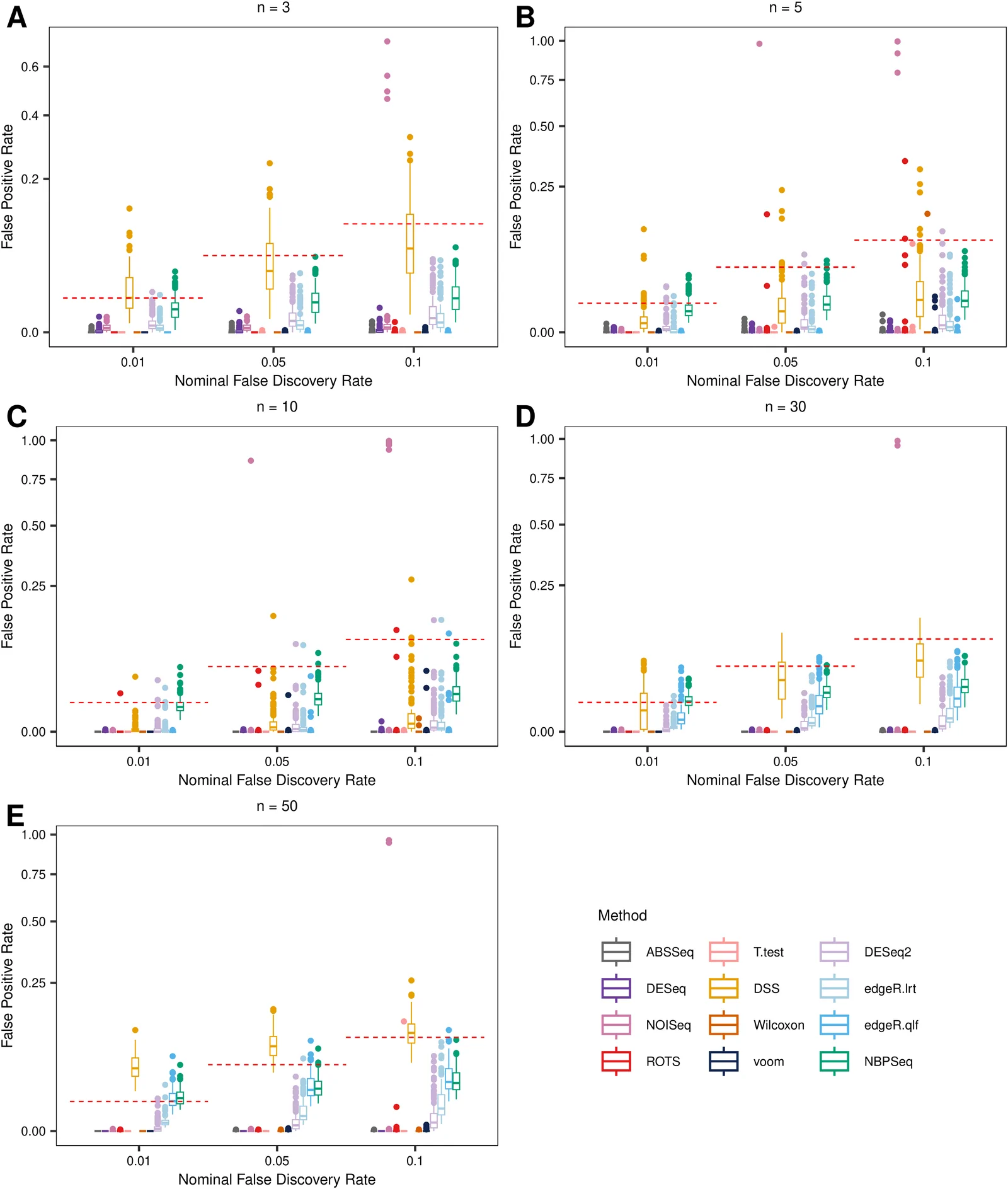
False positive rate (FPR) of DE methods in SCLC dataset of different sample sizes. A) FPR results with a sample size of *n* = 3. Each plot shows the actual FPR when the nominal FDR is set to 0.01, 0.05, and 0.1. The red dashed lines indicate the corresponding expected FPR values. B) Same as A), but with a sample size of *n* = 5. C) Same as A), but with a sample size of *n* = 10. D) Same as A), but with a sample size of *n* = 30. E) Same as A), but with a sample size of *n* = 50.

Overall, the FPR showed a decreasing trend as sample size increased (Fig 12). ABSSeq, DESeq, ROTS, the T.test, voom, and Wilcoxon consistently demonstrated more conservative behavior across all sample sizes. In contrast, edgeR.lrt, NBPSeq, DESeq2, and DSS appeared more sensitive. In BRCA, NOISeq showed a relatively high FPR when the sample size was *n* = 3 (Fig 12A), but became increasingly robust as the sample size grew beyond 3 (Fig 12B-E). In SCLC, DSS exceeded the corresponding expected FPR at larger sample sizes, suggesting a risk of FPR inflation (Fig 13D-E).

### Integrated performance “BaGua” map and practical method-selection guidelines

**Fig 14:**
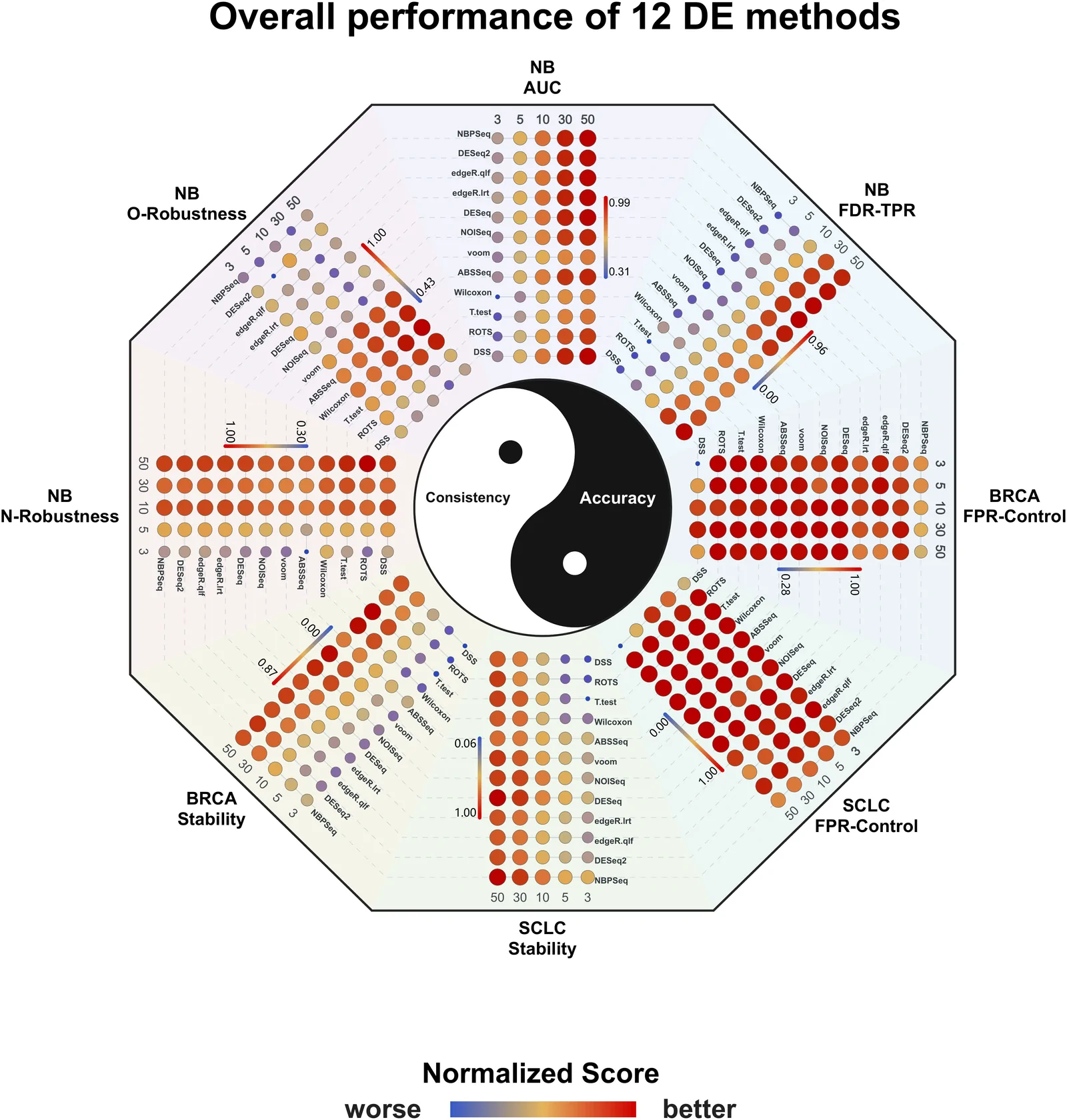
Overall performance of the 12 DE methods across eight evaluation dimensions. The octagonal summary integrates NB simulation accuracy and robustness with stability and false-positive control results from the BRCA and SCLC datasets. Accuracy comprises NB AUC, NB FDR-TPR performance, and BRCA and SCLC FPR control; consistency comprises NB outlier robustness, NB noise robustness, and BRCA and SCLC stability. Bubble color and diameter represent the min-max normalized performance score, with larger red bubbles indicating better performance. Each concentric octagon connects the same DE method across the eight dimensions, and columns within each sector represent sample sizes per group of 3, 5, 10, 30, and 50. Abbreviations used: “Outlier Robustness” = “O”, “Noise Robustness” = “N”.

To integrate results across evaluation dimensions, we converted each method’s performance in simulated and real data analyses into standardized scores and constructed an integrated performance “Bagua” map (Fig 14, S16 Fig in S1 File). The map summarizes relative performance across six dimensions, revealing clear trade-offs: methods performing well in accuracy metrics such as AUC and low-FDR sensitivity did not necessarily perform equally well in consistency metrics such as false-positive control and stability. These findings underscore the scenario dependence of DE-method performance and the limitations of evaluating methods using a single metric.

**Fig 15:**
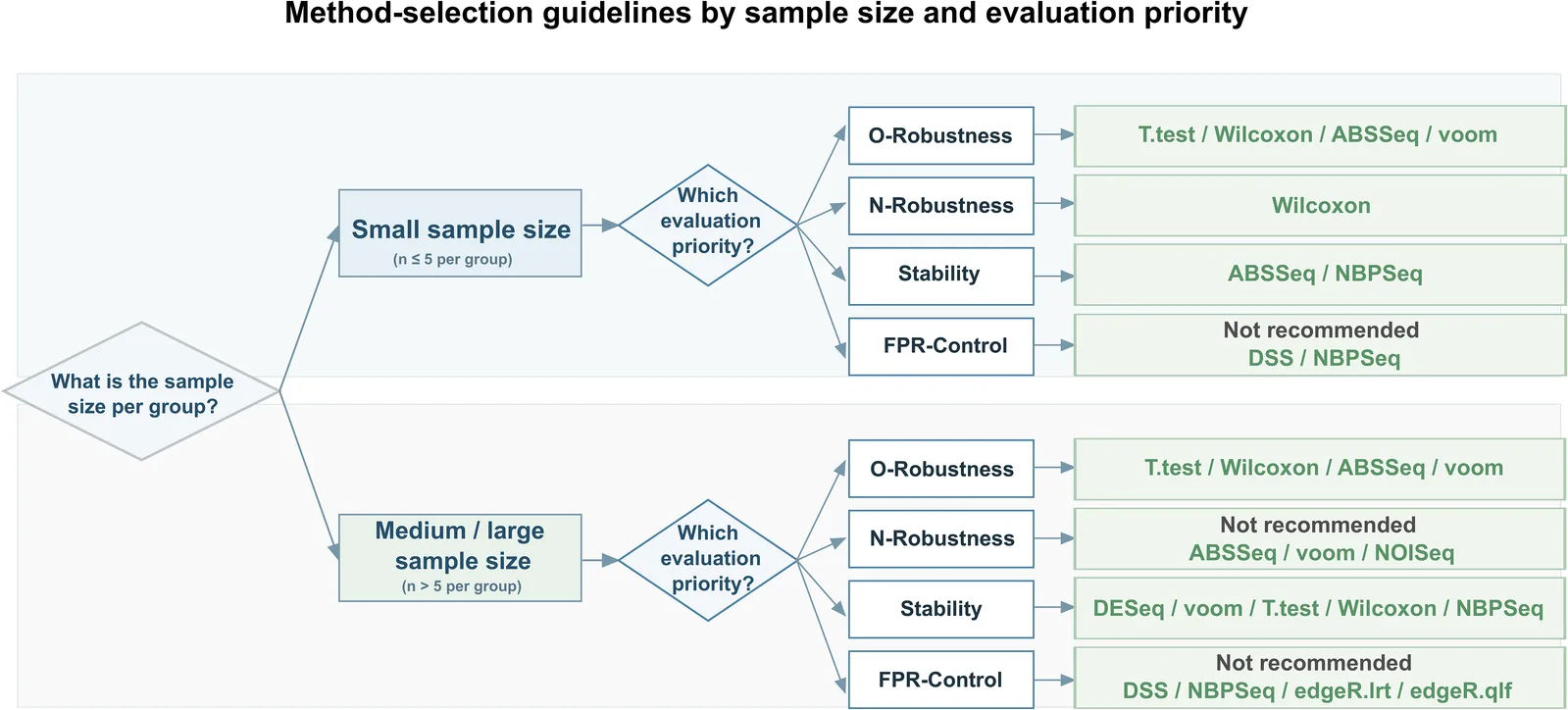
Sample-size-aware guidelines for selecting differential expression methods. The decision framework summarizes recommended DE methods according to sample size per group and the primary evaluation priority. Small-sample settings correspond to *n* = 3 or *n* = 5 per group, whereas medium-to-large sample settings correspond to *n* > 5 per group. Four evaluation priorities are considered: robustness to outliers (O-Robustness), robustness to random noise (N-Robustness), false-positive control (FPR-Control), and stability. Recommended methods indicate those with comparatively favorable performance under each branch, whereas methods shown in smaller gray text indicate comparatively less preferred choices for that specific setting.

We next summarized the benchmark results into sample-size-aware method-selection guidelines (Fig 15). Under small-sample conditions (n≤5), different methods showed advantages depending on the evaluation priority. For outlier robustness, the T.test, Wilcoxon test, ABSSeq, and voom showed relatively favorable performance; for noise robustness, Wilcoxon was preferred; for false-positive control, most methods performed acceptably except DSS and NBPSeq; and for stability, ABSSeq and NBPSeq showed better consistency. For medium-to-large sample sizes (*n* > 5), the preferred methods also depended on the evaluation priority. The T.test, Wilcoxon test, ABSSeq, and voom remained favorable for outlier robustness, whereas most methods performed well for noise robustness except ABSSeq, voom, and NOISeq. For false-positive control, DSS, NBPSeq, edgeR.lrt, and edgeR.qlf were less preferred, whereas DESeq, voom, the T.test, Wilcoxon test, and NBPSeq showed relatively better stability. These recommendations should be regarded as practical guidelines under the evaluated scenarios rather than global superiority rankings.

## Discussion

This study provides a systematic evaluation of multiple RNA-seq DE analysis methods using 80 simulated and real datasets. The simulated datasets offered clear ground truth and flexibility, while the real datasets facilitated the evaluation of false positives and method concordance, thereby incorporating more realistic biological complexity.

Our comparisons focused on six key dimensions of performance: overall detection accuracy, sensitivity under low FDR, false positive rate, robustness to outliers, robustness to noise, and consistency across datasets. These six categories are non-redundant and together provide a more holistic understanding of each method’s performance. While AUC and TPR yielded similar overall outcomes, their inherent differences offered complementary perspectives for performance evaluation. For instance, when sample sizes ≤5, NBPSeq exhibited a high AUC but performed relatively poorly on FDR–TPR curves. Moreover, robustness to noise and robustness to outliers capture distinct dimensions of real-world data variation, reflecting fluctuations in variance and deviations from assumed distributions, respectively. For example, we observed that although voom was robust to outliers (consistent with previous findings by Soneson et al. [45]), its performance deteriorated notably under increased noise conditions. Robustness and stability also reflect different aspects of methodological generalizability across datasets. The false positive rate, on the other hand, is directly related to result specificity and is therefore of particular concern in practical applications. Beyond NB simulations, we also included Poisson simulations without the over-dispersion assumption. These two set of simulations exhibited generally similar trends, emphasizing the robustness of the evaluation.

Throughout our benchmarking, no single method demonstrated superiority across all metrics and sample sizes. For example, ABSSeq exhibited strong robustness to outliers, ROTS showed stronger resistance to random noise at larger sample sizes, and the T.test and voom showed higher stability at large sample sizes. The overall behavior of DESeq2 and the two edgeR algorithms was generally similar, corroborating the findings of Assefa et al. [28]. Although edgeR.lrt and DESeq2 consistently demonstrated high detection accuracy across a range of sample sizes, both methods tended to be non-conservative in controlling the false positive rate, in line with prior studies [29,46]. This trade-off between statistical power and false-positive control can largely be attributed to the distinct statistical strategies adopted by each method. edgeR.lrt employs a likelihood ratio test, which may underestimate uncertainty in dispersion estimation. In addition, it uses an empirical Bayes approach to shrink gene-wise dispersions toward a trended value, thereby enhancing statistical power but also increasing false positives. DESeq2, in contrast, models the relationship between gene mean expression and dispersion using a fitted smooth curve and estimates dispersion by combining this trend with gene-specific values, applying moderation to both dispersion and fold-change estimates. Compared with DESeq2, DESeq demonstrates better validity, albeit with slightly reduced sensitivity at low FDR thresholds. Specifically, DESeq exhibits more stringent control of false positives, a characteristic consistent with findings from multiple previous studies. This behavior may stem from the DESeq strategy of estimating dispersion by selecting the larger value between the gene-specific estimate and the mean-dependent trend. We also observed that DESeq displayed greater stability as sample size increased, suggesting potential benefits for reproducibility. Given the unique strengths and weakness of each method, it might be a plausible way to combine multiple methods in ensemble frameworks, as already implemented in metaseqR2 [47] and scDEA [48] for bulk and single-cell data, respectively. Besides, we observed that the choice of DE method significantly affects downstream enrichment analyses, underscoring the critical role of method selection in DE studies. Based on these findings, we constructed a standardized multidimensional performance map and sample-size-aware method-selection guidelines.

This study has several limitations. First, we focused on two-group comparisons in bulk RNA-seq analysis, and more complex designs require further evaluation. Second, although Poisson simulations complemented the NB simulations, neither model fully captures every mechanism underlying real transcriptomic data. The construction of gold-standard real datasets remains warranted for future benchmarking studies. Third, we used the default or recommended normalization and analysis workflows of each package to reflect routine practice. Systematic exploration of the full parameter space would further unlock the potential of each method.

## Conclusion

In conclusion, this study presents an updated multidimensional benchmark of representative bulk RNA-seq DE methods using complementary simulated and real-data evaluations. The results highlight that the performance of DE methods is context-dependent, and method selection should consider sample size, detection sensitivity, false-positive control, stability, and robustness rather than relying on a single overall metric. The integrated “BaGua” performance map and practical selection guidelines provide a concise reference for choosing appropriate DE methods in different analytical scenarios.

## Supporting information

S1 File Supplementary figures (S1–S16 Figs). This file contains all supporting figures for the differential expression method benchmarking study, including results from negative-binomial simulations, Poisson simulations, and real-data benchmarks.
(PDF)

## Abbreviations

AUC: Area Under Curve
DE: Differential Expression
DEG: Differential Expression Gene
FDR: False Discovery Rate
FPR: False Positive Rate
NB: Negative Binomial
RNA-Seq: (High-throughput) Sequencing of RNA
ROC: Receiver Operating Characteristic
TPR: True Positive Rate
CPM: Count Per Million
QL: Quasi-likelihood
TCGA: The Cancer Genome Atlas
LUSC: Lung Squamous Cell Carcinoma
TCGA-BRCA: The Cancer Genome Atlas-Breast Cancer
TMM: Trimmed Mean of M values
SCLC: Small-cell lung cancer dataset providing by Qian Liu and Hu Zhou
MAD: Median absolute deviation
