# Supplementary material for: Revisiting differential expression analysis: An updated six-dimensional comparative study
Source: PLoS One. 2026 Aug 26;21(8):e0344709. doi: 10.1371/journal.pone.0344709 (PMC13515022; doi:10.1371/journal.pone.0344709)
Supplement: Supplementary file 1 [file pone.0344709.s001.pdf]

Supplementary data for "Revisiting differential expression analysis: an updated six-dimensional comparative study"

Jianxiong Wu[1,2¶], Shaoke Lu[1¶], Hui Yao[1¶], Zhaoyuan Fang[1,2*]

[1] Department of Colorectal Surgery and Oncology, The Second Affiliated Hospital, Zhejiang University School of Medicine, Centre of Biomedical Systems and Informatics, Zhejiang University-University of Edinburgh Institute (ZJU-UoE Institute), Zhejiang University, Hangzhou, Zhejiang, China

[2] Edinburgh Medical School, College of Medicine and Veterinary Medicine, The University of Edinburgh, Edinburgh, United Kingdom

* Corresponding author
E-mail: fangzhaoyuan@sibs.ac.cn; zhaoyuanfang@intl.zju.edu.cn (ZF)

¶ These authors contributed equally to this work.

This document includes Supplementary Figures S1-S16.

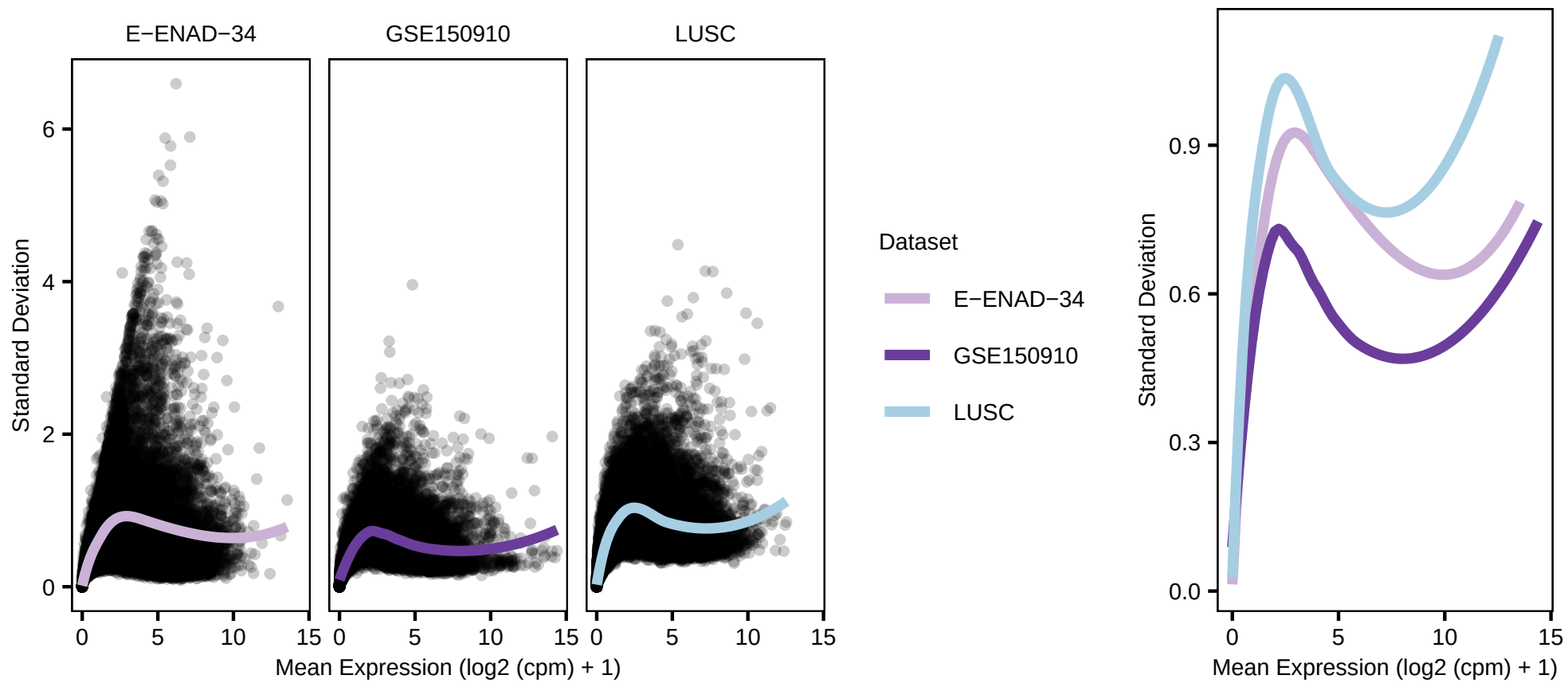

Fig. S1 Standard deviation against mean expression ($\log_2(\text{CPM} + 1)$) for the original real datasets, with Loess smoothing curves showing the relationship between expression variability and expression level.

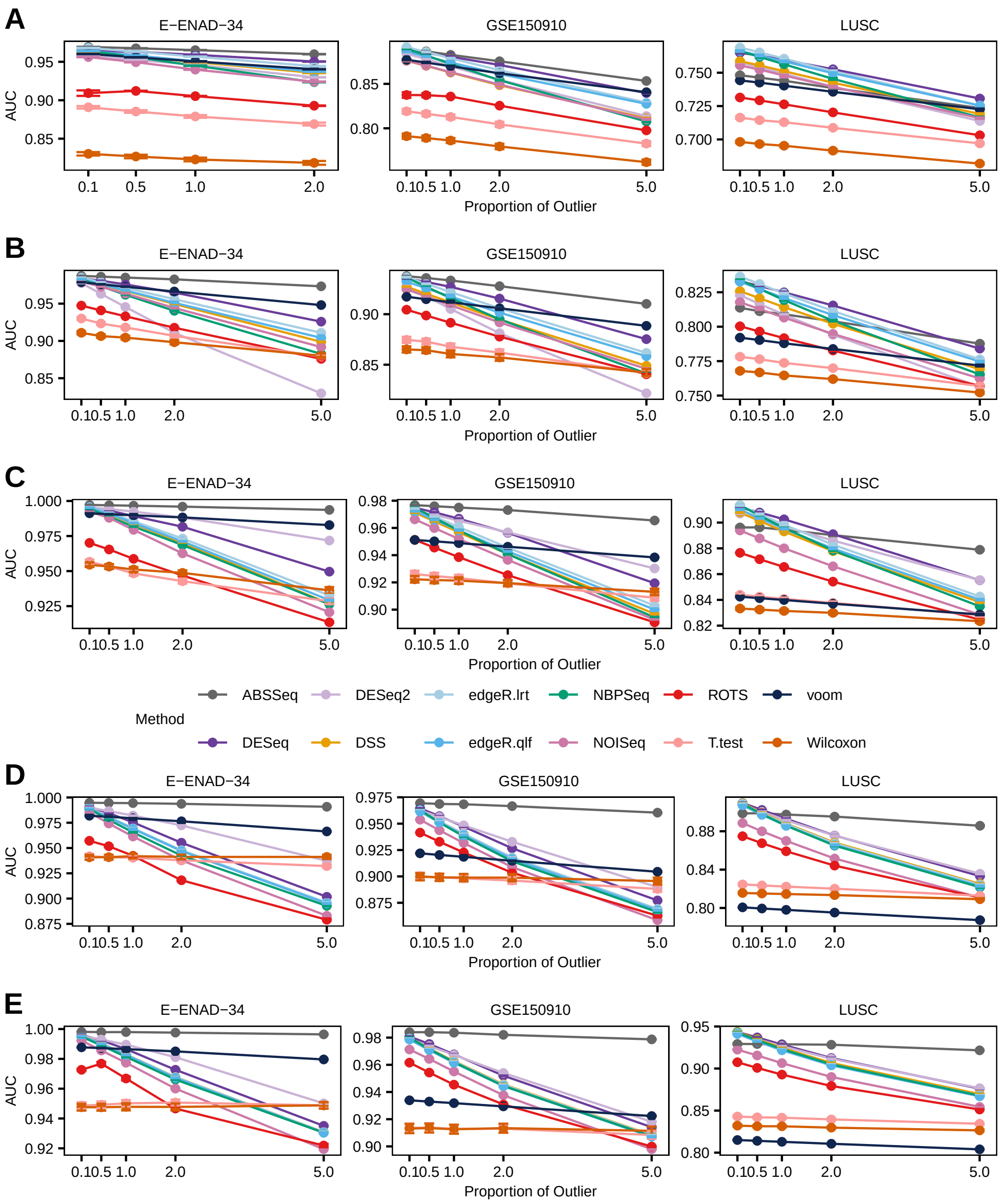

Fig. S2 Robustness of DE methods in negative-binomial simulations with outliers of different sample sizes.

A) Systematic analysis of AUC changes for different methods under varying outlier proportions in three types of simulated datasets with a sample size of 3. Each boxplot is based on 100 simulation iterations. Outlier proportions are set to 0.1%, 0.5%, 1%, 2%, and 5%. For instance, an outlier proportion of 0.1% means that 0.1% of the count values were multiplied by a random number uniformly sampled from the interval [0, 10], and then rounded to the nearest integer.
B) Same as A), but with a sample size of 5.
C) Same as A), but with a sample size of 10.
D) Same as A), but with a sample size of 30.
E) Same as A), but with a sample size of 50.

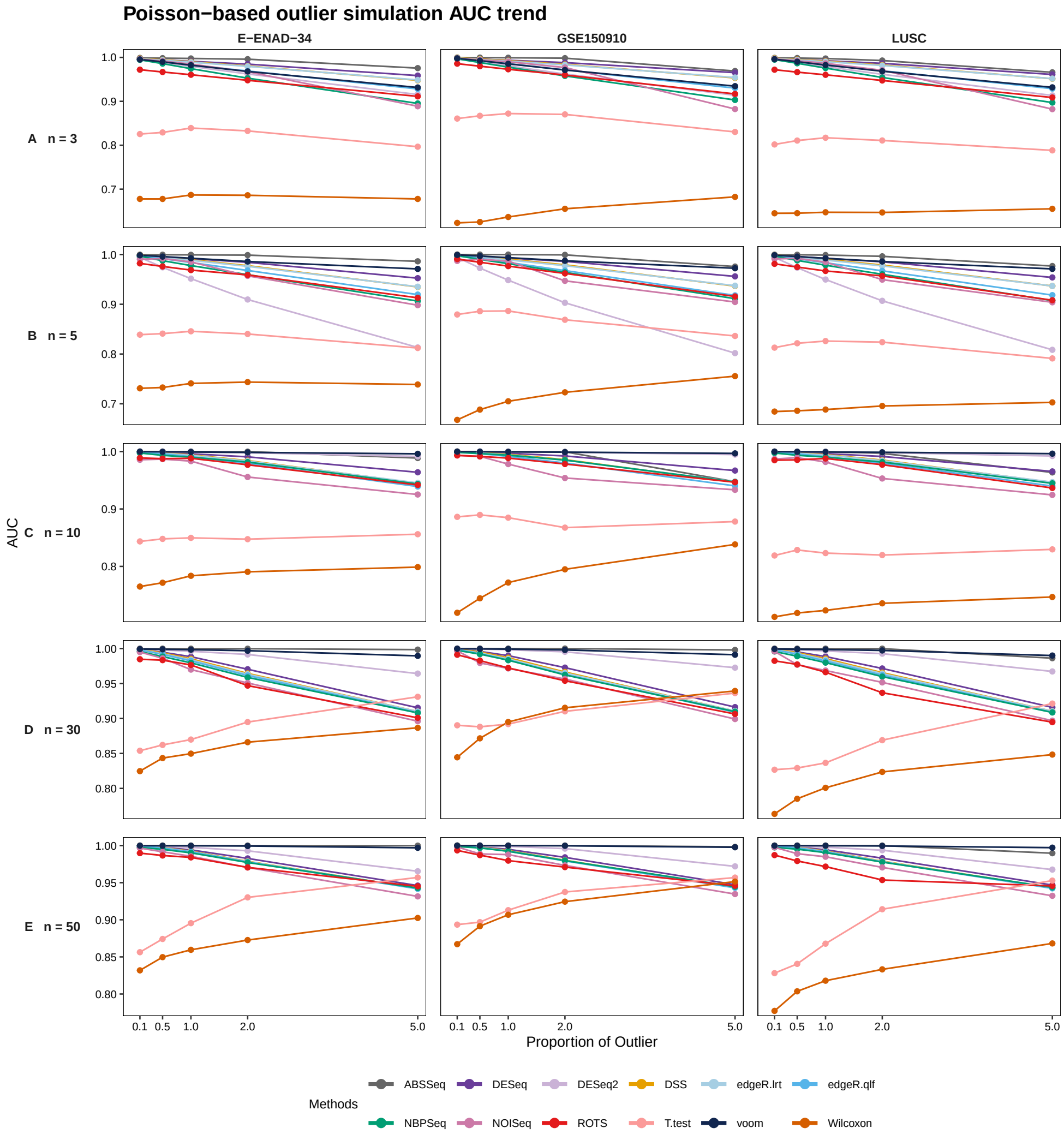

Fig. S3 Robustness of DE methods in negative-binomial simulations with noises of different sample sizes.

A) Systematic analysis of AUC changes for different methods under varying outlier proportions in three types of simulated datasets with a sample size of 3. Each boxplot is based on 100 simulation iterations. Outlier proportions are set to 0.1%, 0.5%, 1%, 2%, and 5%. For instance, an outlier proportion of 0.1% means that 0.1% of the count values were multiplied by a random number uniformly sampled from the interval [0, 10], and then rounded to the nearest integer.

B) Same as A), but with a sample size of 5.

C) Same as A), but with a sample size of 10.

D) Same as A), but with a sample size of 30.

E) Same as A), but with a sample size of 50.

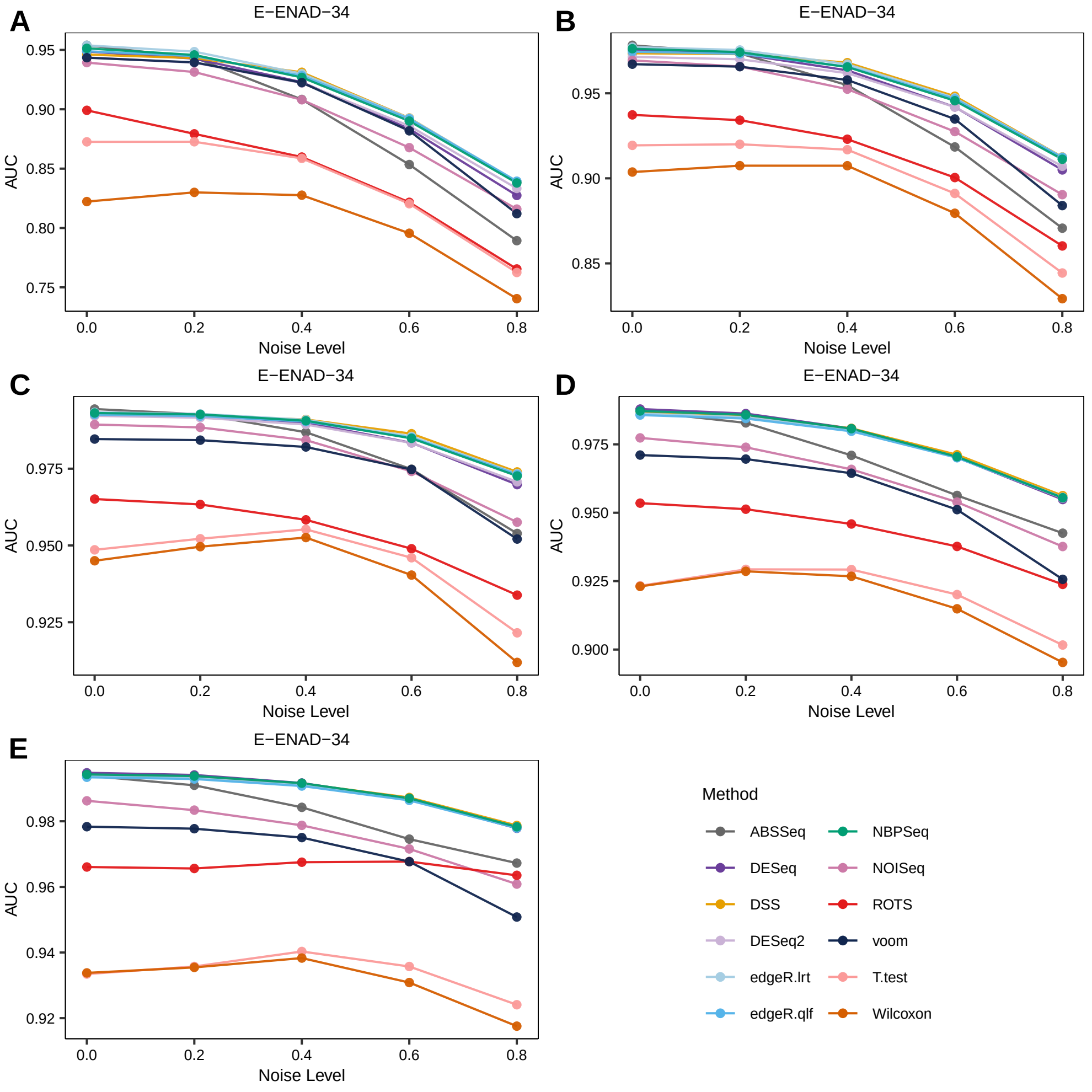

Fig. S4 Robustness of DE methods to noise in negative binomial simulations of different sample sizes. A) AUC changes of different DE methods under varying noise levels with a sample size of 3. Each boxplot is based on 100 simulation iterations. Noise levels include 0.2, 0.4, 0.6, and 0.8.
B) Same as A), but with a sample size of 5.
C) Same as A), but with a sample size of 10.
D) Same as A), but with a sample size of 30.
E) Same as A), but with a sample size of 50.

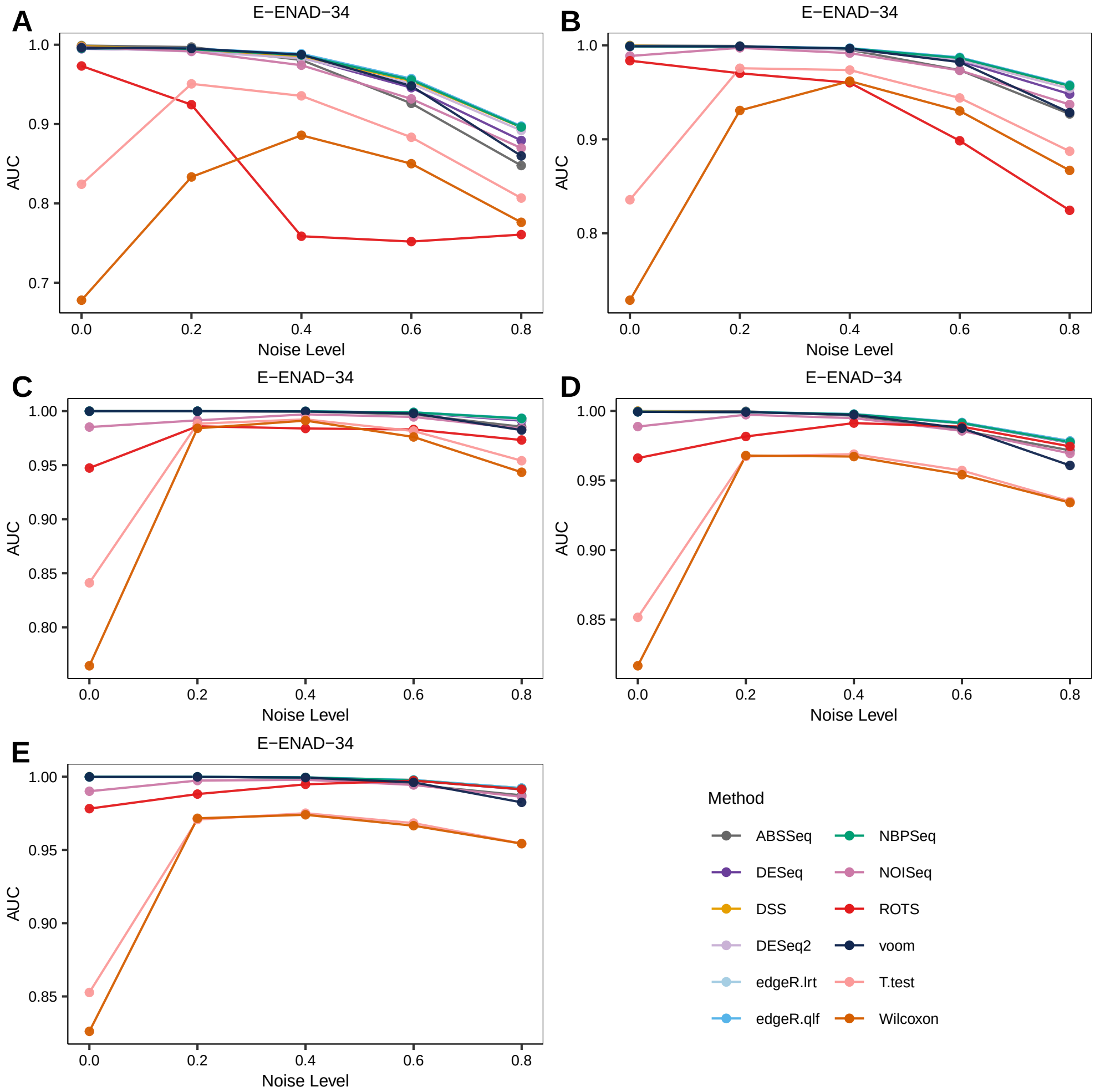

Fig. S5 Robustness of DE methods to noise in poisson simulations of different sample sizes. A) AUC changes of different DE methods under varying noise levels with a sample size of 3. Each boxplot is based on 100 simulation iterations. Noise levels include 0.2, 0.4, 0.6, and 0.8.
B) Same as A), but with a sample size of 5.
C) Same as A), but with a sample size of 10.
D) Same as A), but with a sample size of 30.
E) Same as A), but with a sample size of 50.

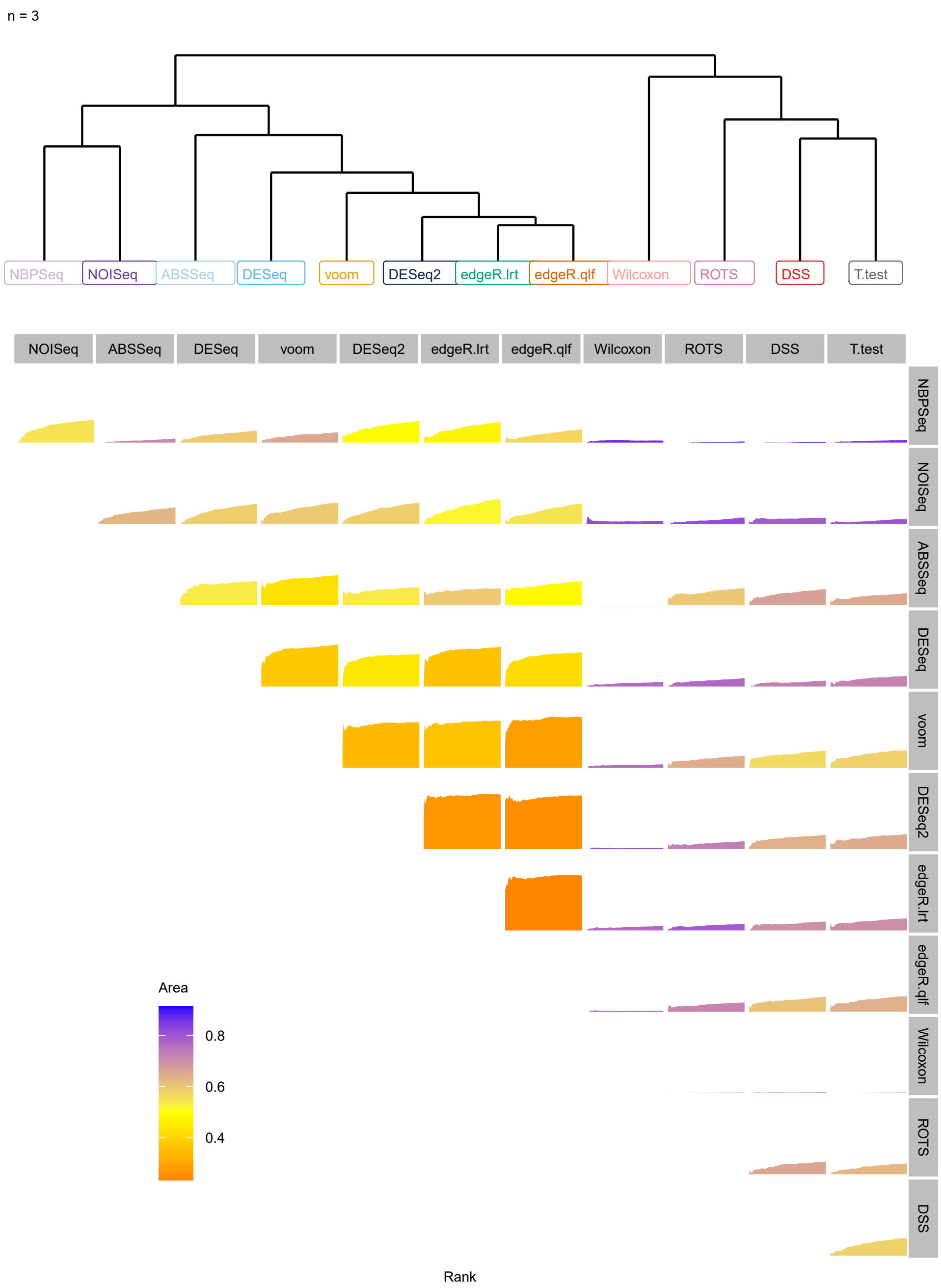

Fig. S6 Similarity of DE methods.The ribbon plot of concordance from rank 1 to the top 10% for DE methods represents the consistency among the methods. The area size was converted into distance to perform hierarchical clustering dendrogram for 11 DE methods. DESeq2, edgeR.lrt and edgeR.qlf cluster into the closest group. The sample size is 3.

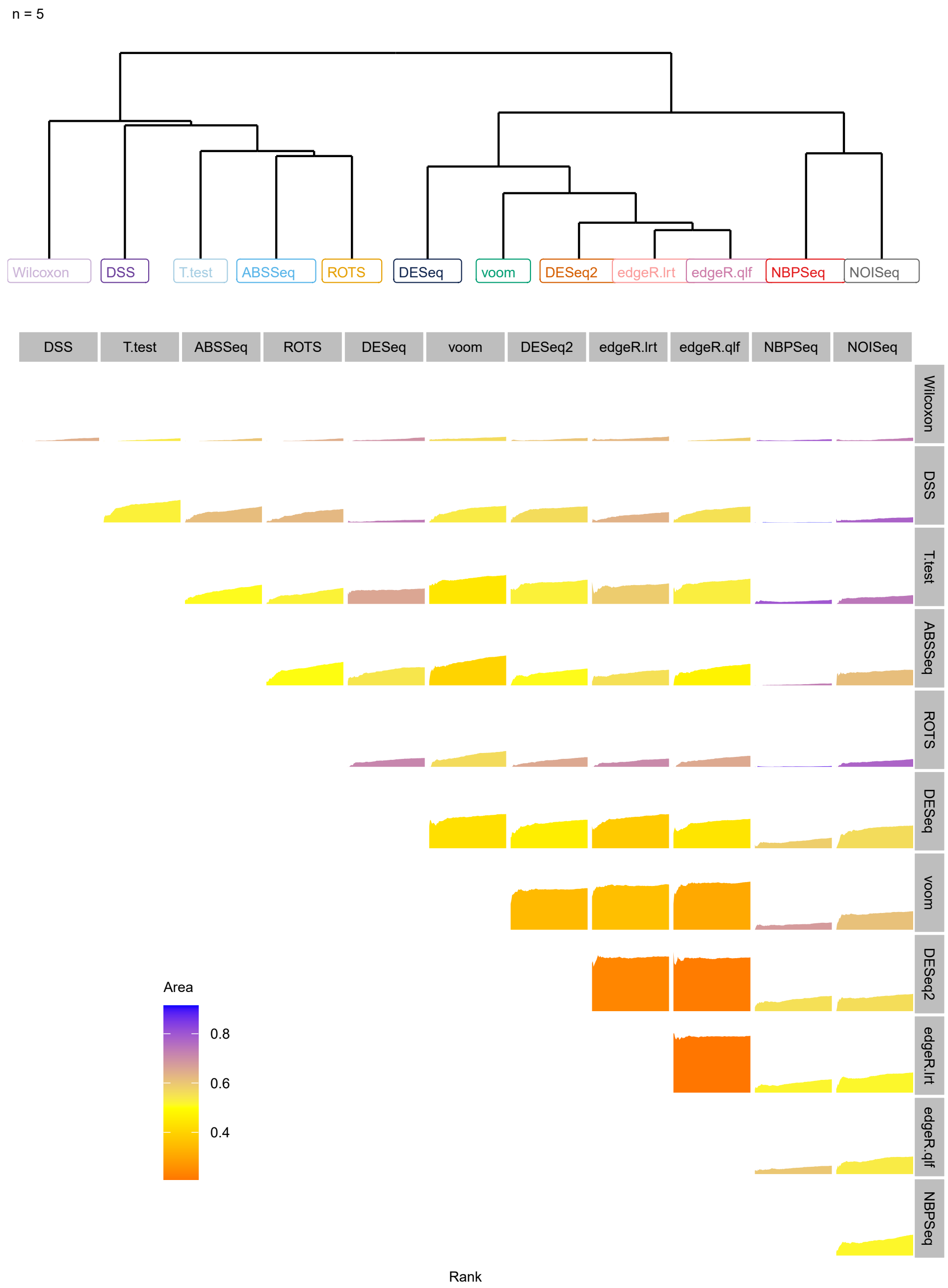

Fig. S7 Similarity of DE methods.The ribbon plot of concordance from rank 1 to the top 10% for DE methods represents the consistency among the methods. The area size was converted into distance to perform hierarchical clustering dendrogram for 11 DE methods. DESeq2, edgeR.lrt and edgeR.qlf cluster into the closest group. The sample size is 5.

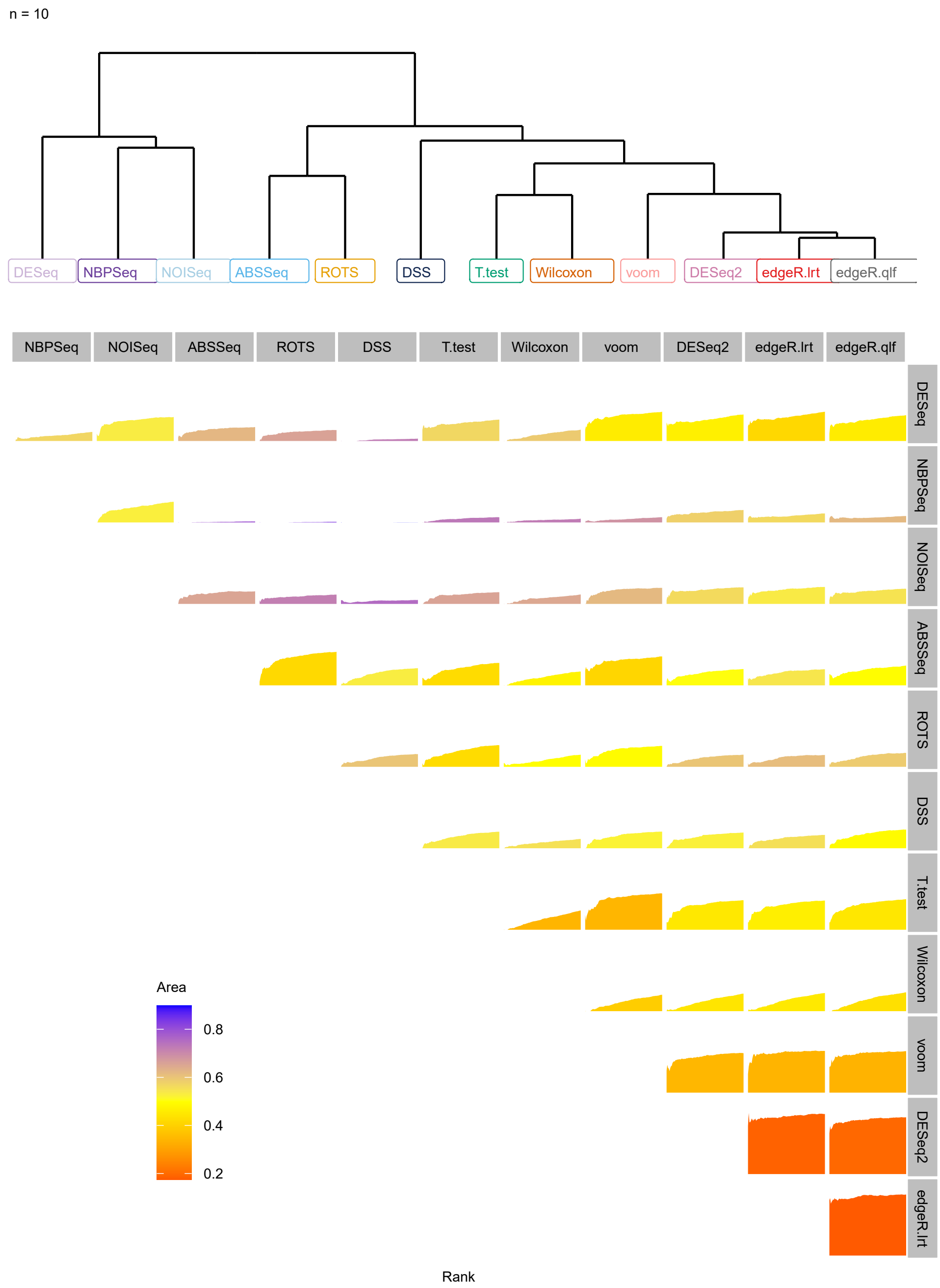

Fig. S8 Similarity of DE methods.The ribbon plot of concordance from rank 1 to the top 10% for DE methods represents the consistency among the methods. The area size was converted into distance to perform hierarchical clustering dendrogram for 11 DE methods. DESeq2, edgeR.lrt and edgeR.qlf cluster into the closest group. The sample size is 10.

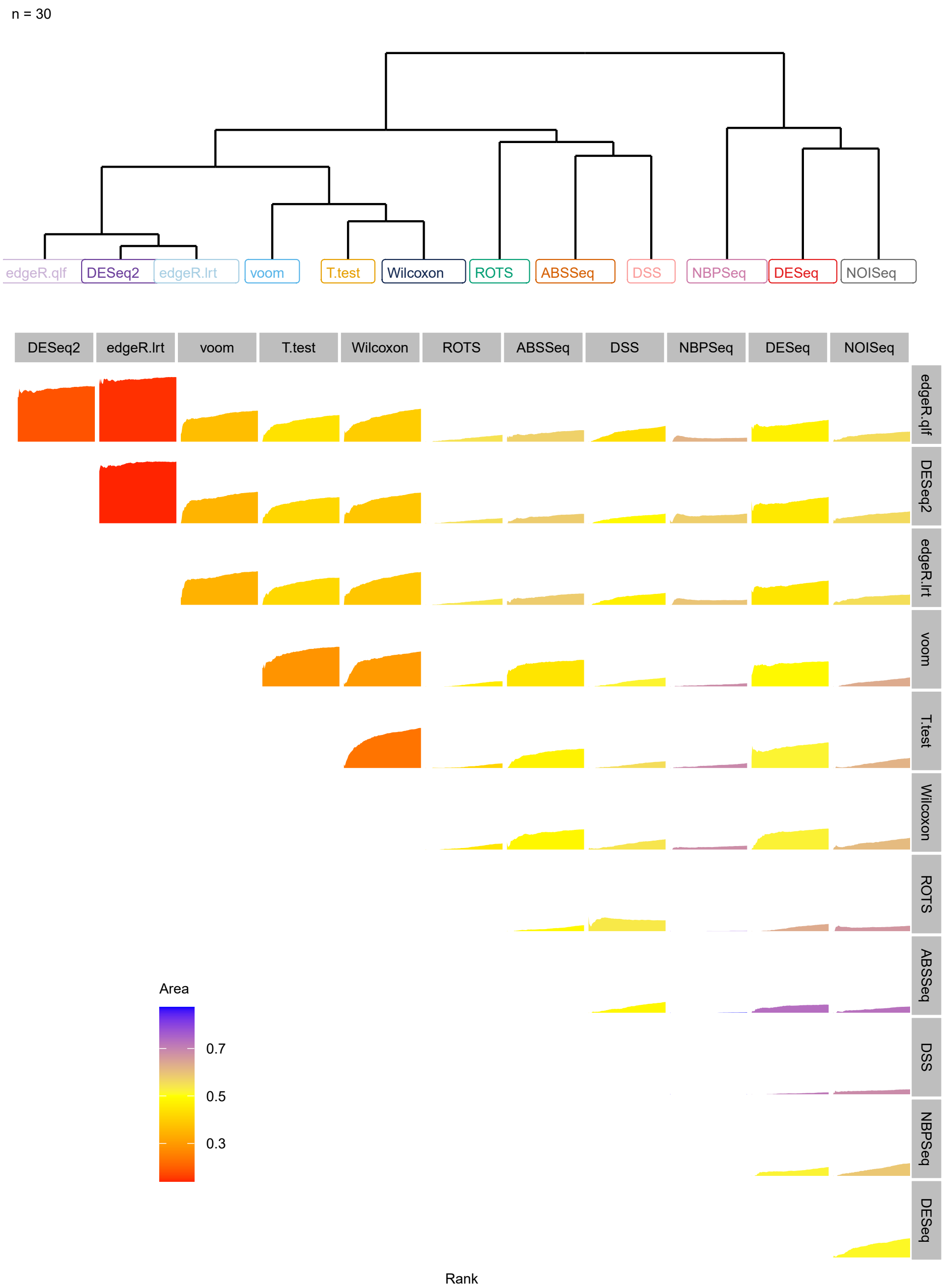

Fig. S9 Similarity of DE methods.The ribbon plot of concordance from rank 1 to the top 10% for DE methods represents the consistency among the methods. The area size was converted into distance to perform hierarchical clustering dendrogram for 11 DE methods. DESeq2, edgeR.lrt and edgeR.qlf cluster into the closest group. The sample size is 30.

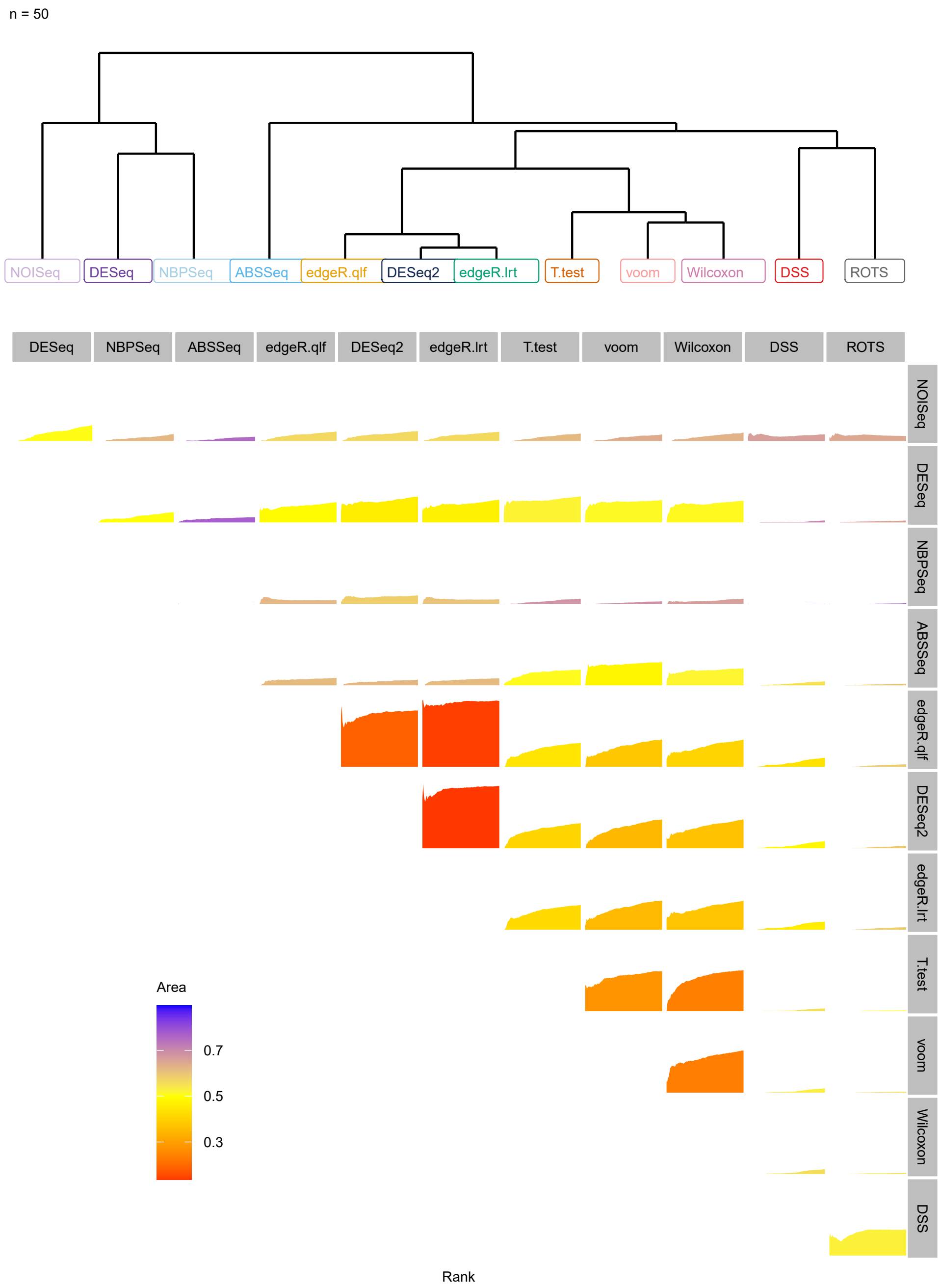

Fig. S10 Similarity of DE methods.The ribbon plot of concordance from rank 1 to the top 10% for DE methods represents the consistency among the methods. The area size was converted into distance to perform hierarchical clustering dendrogram for 11 DE methods. DESeq2, edgeR.lrt and edgeR.qlf cluster into the closest group. The sample size is 50.

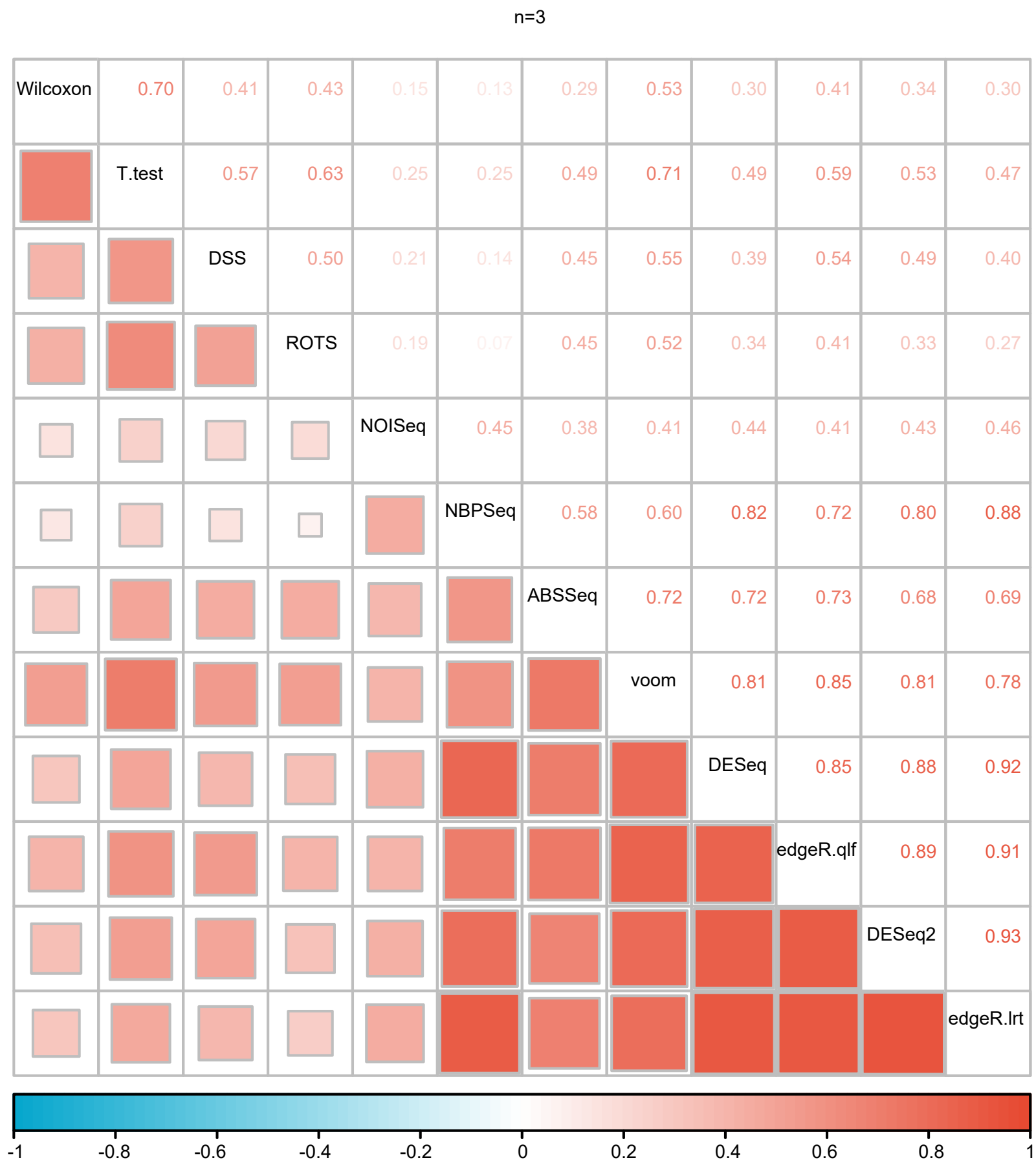

Fig. S11 Similarity of enrichment analysis result for DE methods. Correlation diagram of the results of DE methods of downstream enrichment analysis. The sample size is 3.

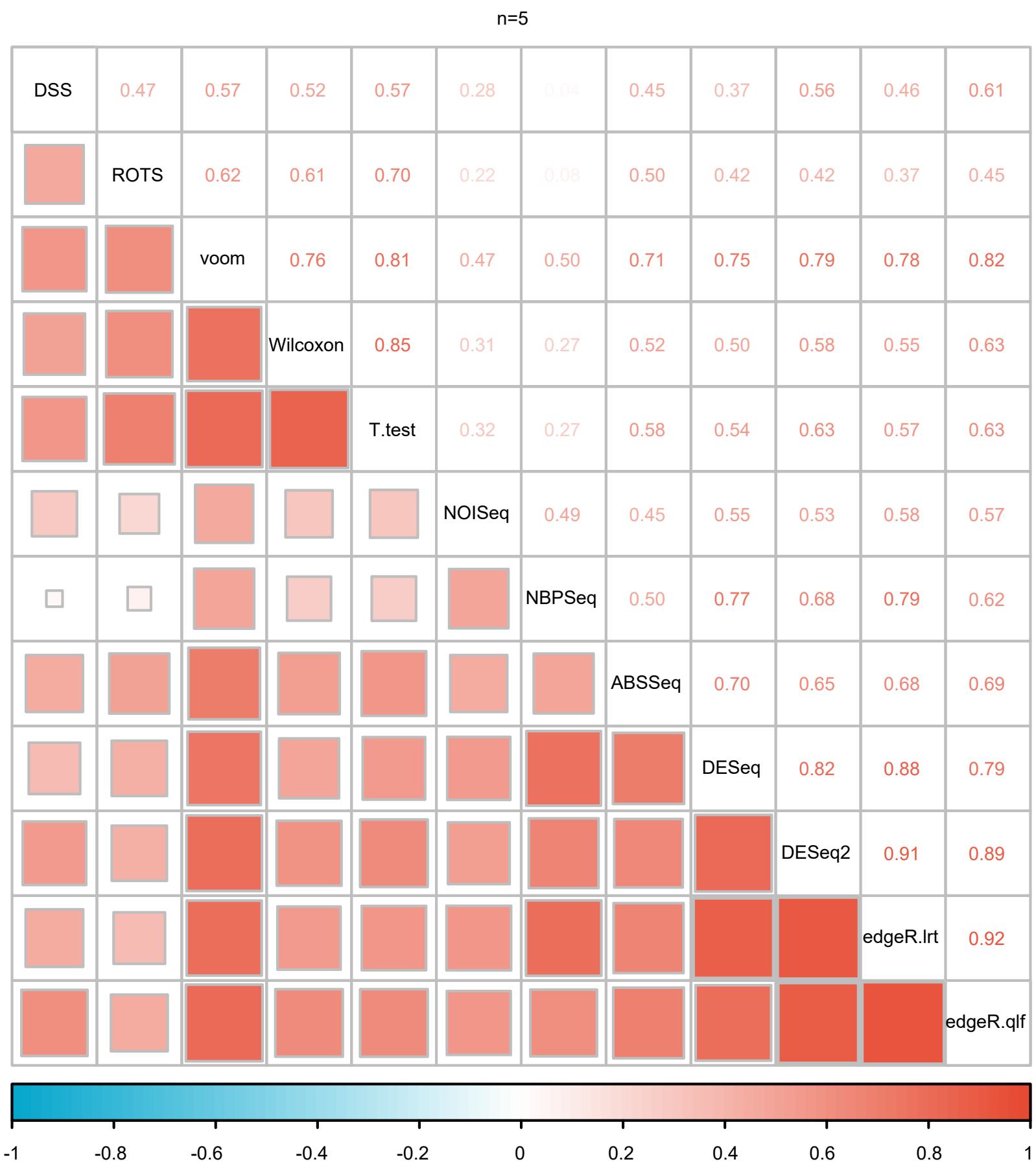

Fig. S12 Similarity of enrichment analysis result for DE methods. Correlation diagram of the results of DE methods of downstream enrichment analysis. The sample size is 5.

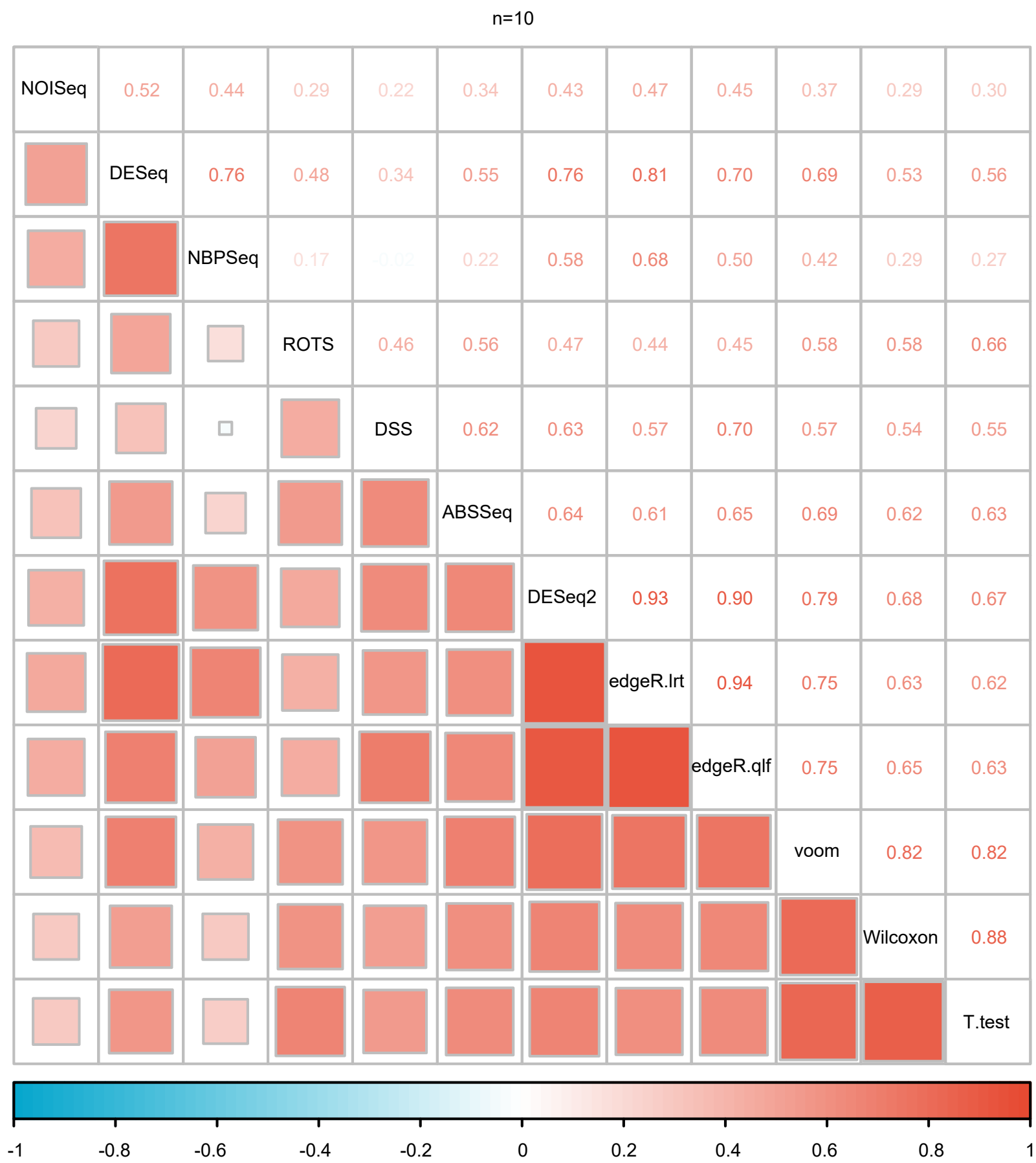

Fig. S13 Similarity of enrichment analysis result for DE methods. Correlation diagram of the results of DE methods of downstream enrichment analysis. The sample size is 10.

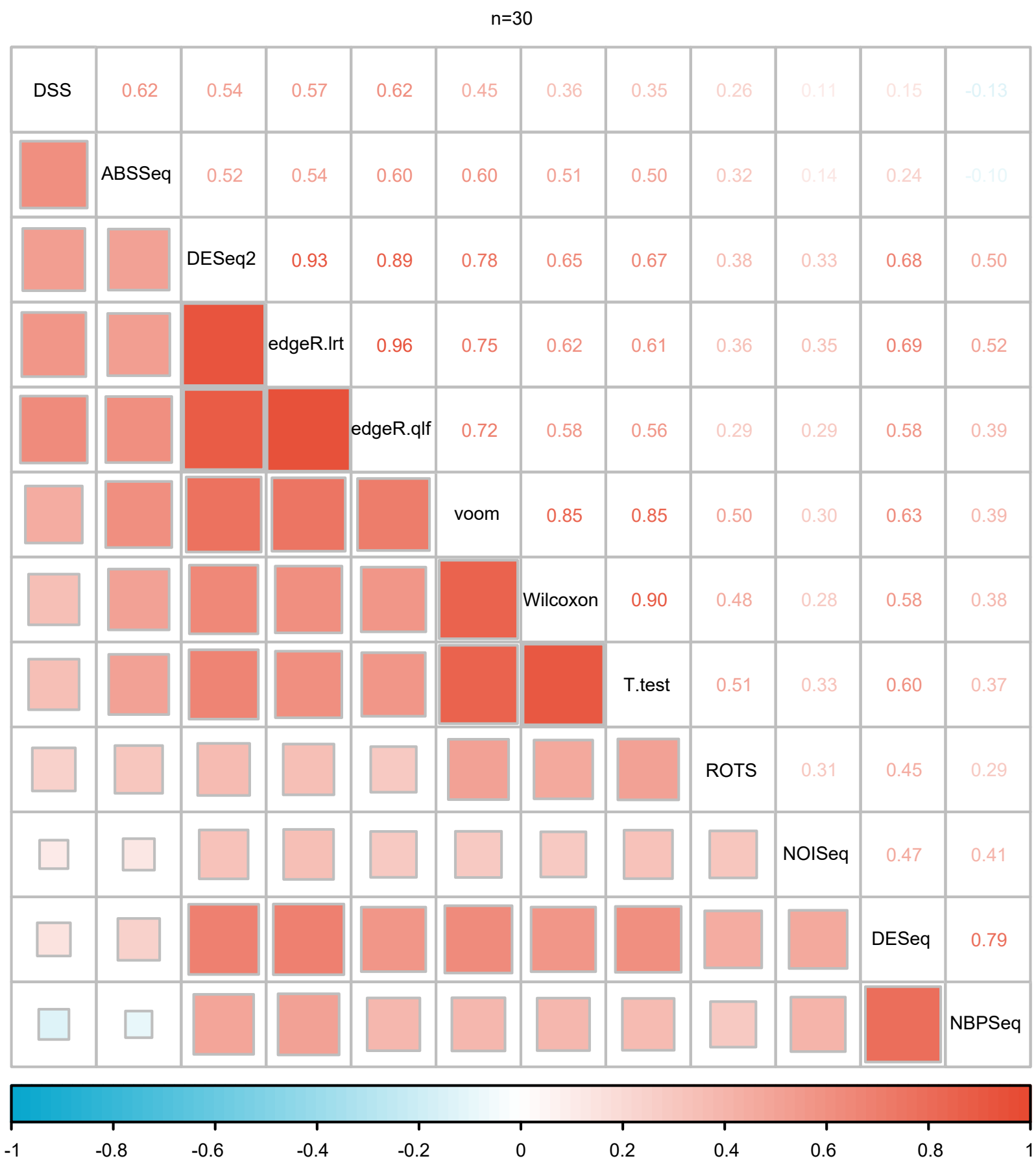

Fig. S14 Similarity of enrichment analysis result for DE methods. Correlation diagram of the results of DE methods of downstream enrichment analysis. The sample size is 30.

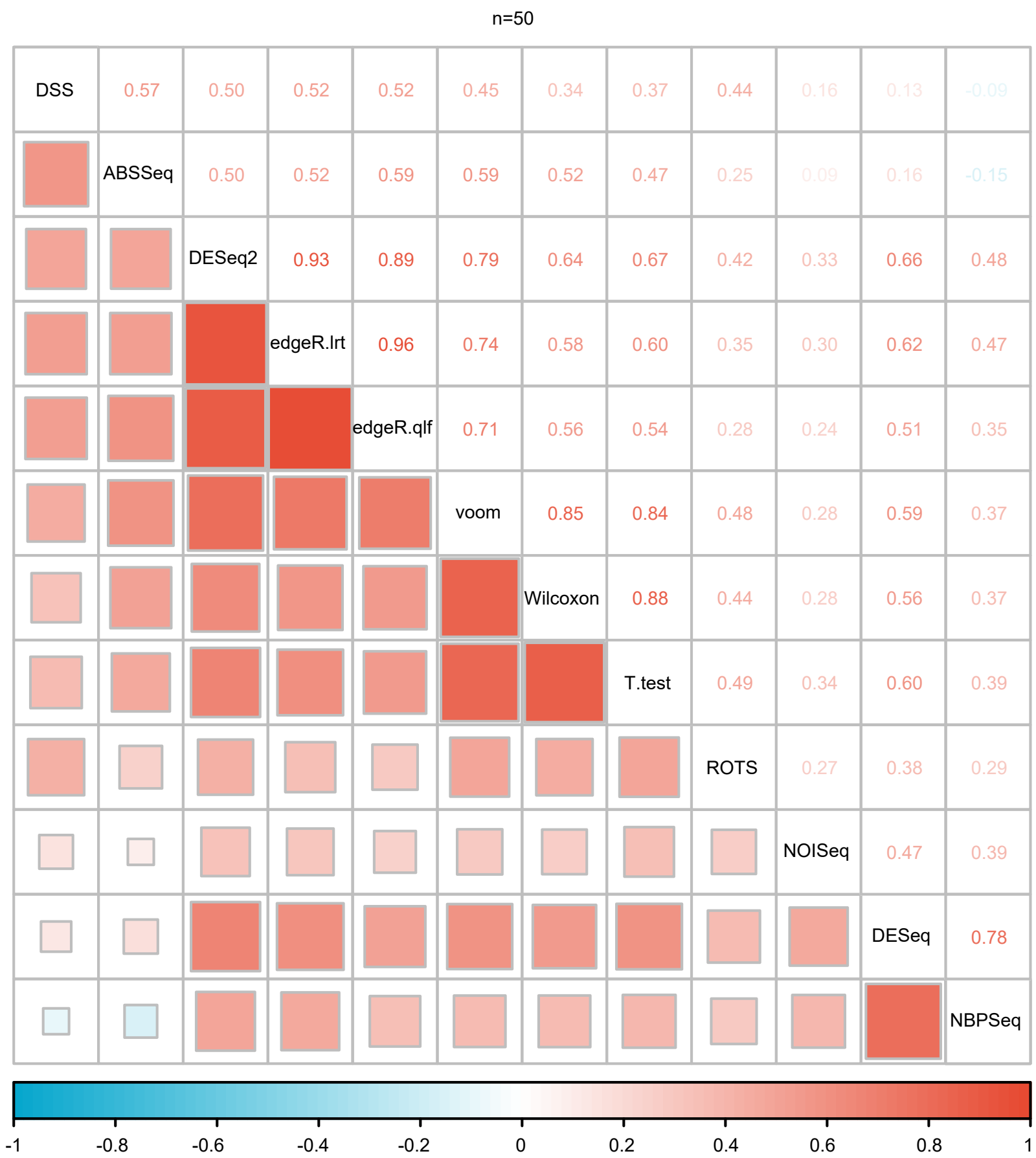

Fig. S15 Similarity of enrichment analysis result for DE methods. Correlation diagram of the results of DE methods of downstream enrichment analysis. The sample size is 50.

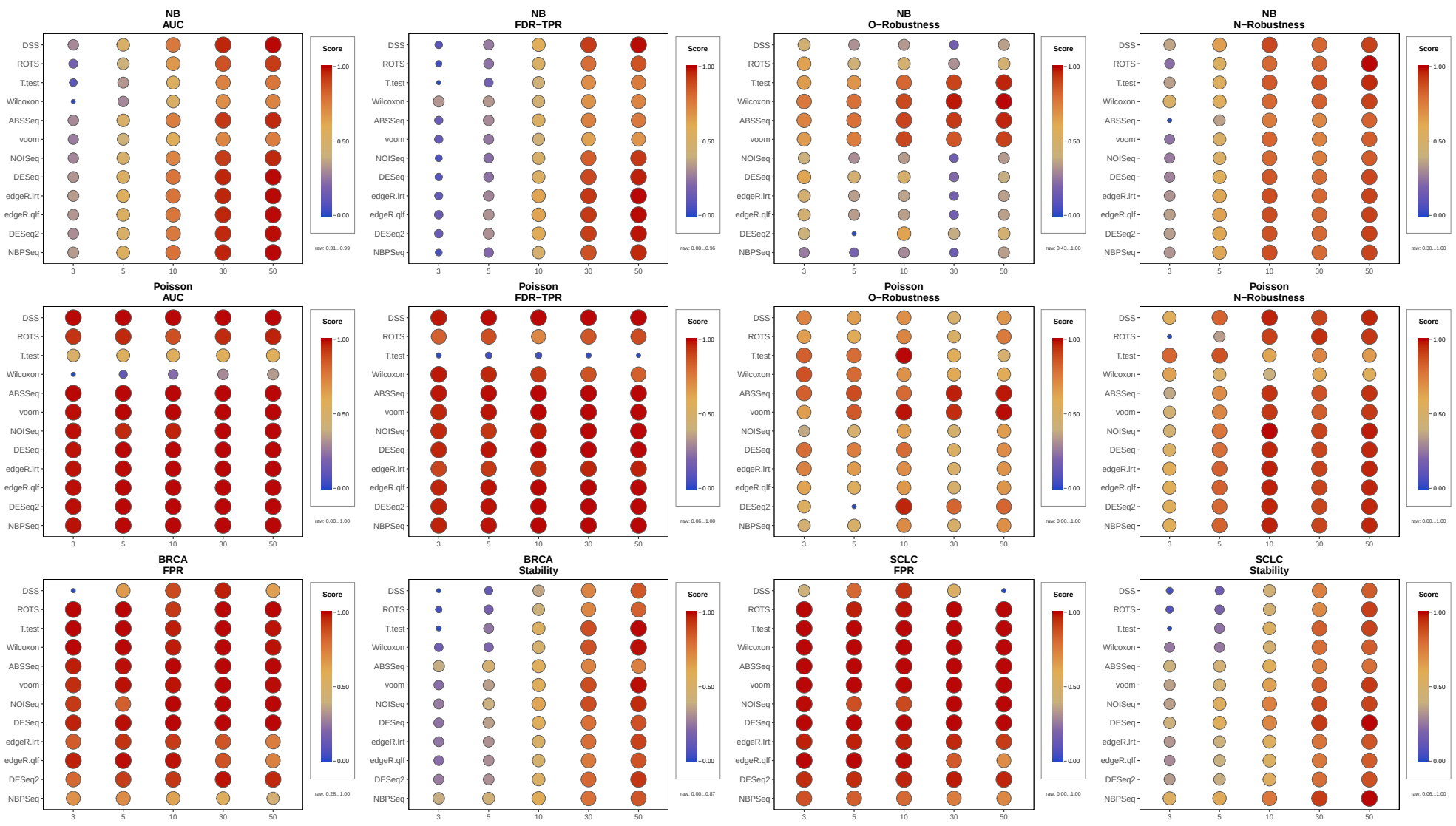

Fig. S16 Multidimensional bubble-map summary of DE method performance.
The bubble plot summarizes the relative performance of 12 DE methods across NB simulations, Poisson simulations, and real-data benchmarks. The 12 panels include AUC, TPR in low FDR region, outlier robustness (O-Robustness), noise robustness (N-Robustness), FPR-control, and stability. The x-axis indicates sample size per group, and the y-axis lists the evaluated methods. Bubble size and color represent internally normalized performance scores within each panel; larger and warmer-colored bubbles indicate better relative performance. All scores were oriented so that higher values indicate more favorable performance. The panel-specific scale bar shows the normalized plotting score from 0 to 1, whereas the raw range indicates the un-re-normalized score range before internal visual scaling. Because scores were internally normalized within each panel, the bubble size and color should be interpreted as relative performance within the same evaluation dimension, not as absolute values directly comparable across different panels.
